# Trends in the disease burden of maternal sepsis and other maternal infections attributable to iron deficiency from 1990 to 2021 and its projection until 2050

**DOI:** 10.3389/fpubh.2025.1658505

**Published:** 2025-09-04

**Authors:** Chunfeng Zhu, Zhiyuan Yang, Haiyun Yuan, Chunyan Yang

**Affiliations:** ^1^Department of Obstetrics, Guangdong Provincial People's Hospital (Guangdong Academy of Medical Sciences), Southern Medical University, Guangzhou, China; ^2^School of Clinical and Basic Medicine, Shandong First Medical University & Shandong Acadamy of Medical Sciences, Jinan, China; ^3^Department of Cardiac Surgery, Guangdong Provincial People's Hospital (Guangdong Academy of Medical Sciences), Southern Medical University, Guangzhou, China; ^4^Guangdong Cardiovascular Institute, Guangdong Provincial People's Hospital, Guangdong Academy of Medical Sciences, Guangzhou, China; ^5^Guangdong Provincial Key Laboratory of South China Structural Heart Disease, Guangzhou, China

**Keywords:** maternal sepsis, maternal infections, iron deficiency, global burden of disease, trends, predication

## Abstract

**Background:**

Iron deficiency is a key risk factor for maternal sepsis and other maternal infections (MSMIs). This study aims to investigate the trends of MSMIs attributable to iron deficiency based on data from the Global Burden of Disease (GBD) 2021 database.

**Methods:**

Data on MSMIs attributable to iron deficiency were utilized. Epidemiological tendencies were assessed using the estimated annual percentage change (EAPC) for age-standardized rate in mortality (ASMR) and disability-adjusted life years (DALYs). The correlation between socio-demographic index (SDI) and age-standardized rates (ASRs) of deaths and DALYs in different GBD regions was evaluated using the Pearson correlation coefficient. Bayesian age-period-cohort models were applied to predict the burden of MSMIs attributable to iron deficiency by 2050.

**Results:**

From 1990 to 2021, the global mortality and DALYs of MSMIs attributable to iron deficiency declined, with an EAPC of −2.49 (95% confidence interval (CI): −2.91 to −2.06) and −2.51 (95% CI: −2.92 to −2.10), respectively. The decrease of ASMR was the most in the high-middle SDI regions, while for age-standardized DALY rate, the most decrease was in the low-middle SDI regions. In 2021, Somalia represented the greatest burdens on deaths and DALYs, with an ASMR of 2.98 per 100,000 persons (95% uncertainty interval (UI): 1.30–5.12) and an age-standardized DALY rate of 177.38 per 100,000 persons (95% UI: 76.95–299.71). East Asia had the most decreases in the burdens of deaths and DALYs. Moreover, Equatorial Guinea represented the largest decreases in ASMR and age-standardized DALY rate. In 1990 and 2021, the peaks of MSMIs-related deaths (1,124 cases and 724 cases) and DALYs (82,593 cases and 53,355 cases) were found in the 20–24 years age group. The ASMR and age-standardized DALY rate of MSMIs attributable to iron deficiency were negatively correlated with SDI. Projections indicated a continued decrease in the burden of MSMIs attributable to iron deficiency by 2050.

**Conclusions:**

Over the past 30 years, the global burden of MSMIs attributable to iron deficiency has been decreasing, which is predicted to continually decrease by 2050. Targeted strategies for improving management in MSMIs attributable to iron deficiency should focus on 20–24 years population.

## 1 Introduction

Maternal health is one of global public health issues, with infectious diseases (sepsis, urinary tract infections, and postpartum infections) being significant contributors to maternal mortality (1–3). In addition to the factors of sanitation and the health status of pregnant women, maternal sepsis and other maternal infections (MSMIs) are typically caused by bacterial infections, with the major pathogens including Listeria monocytogenes, Escherichia coli, and Staphylococcus aureus (4, 5). Bacterial pathogens entering the maternal bloodstream trigger inflammatory immune responses. However, dysregulated or excessive inflammation can cause tissue damage and organ failure, characterizing sepsis (6). Epidemiological data indicate that in 2021, the global incidence of MSMIs reached 19.05 million cases (7). The occurrence of MSMIs is associated with multiple factors, including known risk factors of immunocompromised status, postpartum complications, and infections during labor. Recent report suggests that iron deficiency is also a significant risk factor for MSMIs (7). Iron is an essential trace element in the human body, playing critical roles in oxygen transport, DNA synthesis, and immune system function (8). Iron is essential for the proliferation, differentiation, and effector functions of immune cells. Iron deficiency impairs the functions of macrophages, T cells, and B cells, leading to a weakened immune response, which makes pregnant women more susceptible to bacterial and viral infections (8, 9). According to the World Health Organization (WHO), ~30% of the global population suffers from iron deficiency anemia, and iron deficiency is one of the primary causes of anemia during pregnancy (10). However, the global impact of iron deficiency on MSMIs has not been fully explored.

In recent years, the Global Burden of Disease (GBD) study has emerged as one of the most impactful international collaborative research initiatives, with the goal of offering robust evidence to inform global health policy decisions through a comprehensive analysis of the global burden of MSMIs disease (7, 11). According to the GBD 2019 data, the age-standardized rates (ASRs) of incidence and mortality for MSMIs in 2019 are 1,072.90 per 100,000 persons and 0.86 per 100,000 persons, respectively (11). Furthermore, the latest GBD 2021 data show that the global incidence of MSMIs has steadily decreased from 22 million cases in 1990 to 12 million cases in 2021 (7). There are significant regional differences in the burden of MSMIs, depending on the socio-demographic index (SDI). The GBD 2021 study indicates that the disease burden of MSMIs has significantly decreased in the high SDI regions, while it has increased in the low SDI regions (7). Notably, iron deficiency is a critical risk factor for MSMIs, particularly in regions with lower SDI level (7). These regions exhibit higher prevalence of iron deficiency, compounded by limited public health resources, which pose greater challenges to maternal health. While previous GBD studies have documented the overall burden and trends of MSMIs (7, 11), the specific contribution and temporal patterns of iron deficiency -attributable MSMIs remain underexplored at the global level. A recent GBD 2021 analysis highlighted regional disparities in MSMI burden linked to SDI and identified iron deficiency as a critical risk factor, particularly in low-resource settings (7). However, a comprehensive assessment focusing specifically on the iron deficiency-attributable fraction of MSMI burden, its trends across different geographies and age groups, and future projections is lacking.

Iron deficiency is defined as a state in which there is insufficient iron to meet the body's physiological needs, often measured by low serum ferritin levels (typically < 15 μg/L) or other indicators of depleted iron stores (8, 10). It is distinct from anemia of other etiologies (e.g., vitamin B12/folate deficiency, chronic disease, hemoglobinopathies), although it is the most common cause of anemia globally and during pregnancy (Iron Deficiency Anemia, IDA) (8, 10). Iron deficiency impairs critical immune functions even before frank anemia develops (8, 9). This study focuses specifically on the burden of MSMIs attributable to this defined state of iron deficiency, as quantified within the GBD comparative risk assessment framework.

Based on GBD 2021 data, this study aims to investigate the global, regional, and national burden of MSMIs attributable to iron deficiency from 1990 to 2021, which is to reduce the disease burden attributable to iron deficiency through targeted early intervention. Additionally, a detailed analysis of future trends in MSMIs attributable to iron deficiency was provided. Our study will comprehensively evaluate the role of iron deficiency in the global MSMIs burden, filling a research gap in this field and making a positive contribution to improving global maternal health.

## 2 Methods

### 2.1 Data acquirement

Data on the burden of MSMIs attributable to ID for the period 1990–2021 were extracted from the Global Health Data Exchange (GHDx) query tool (http://ghdx.healthdata.org) of the GBD 2021. The GBD 2021 study provides comprehensive estimates of mortality, disability-adjusted life years (DALYs), and their corresponding ASRs for diseases and injuries globally, incorporating data from a wide array of sources (e.g., vital registration, verbal autopsy, surveillance systems, published literature) and employing standardized estimation methods. Data were obtained at multiple levels: global, 5 SDI quintiles (High, High-middle, Middle, Low-middle, Low), 4 continents, 6 World Health Organization (WHO) regions, 21 GBD super-regions, and 204 countries and territories. Age-specific data were analyzed for seven 5-year age groups (15–19, 20–24, 25–29, 30–34, 35–39, 40–44, and 45–49 years). The SDI, a composite measure of income per capita, average educational attainment, and total fertility rate, ranges from 0 (lowest development) to 1 (highest development) (12). The age was divided into 7 subgroups: 15–19, 20–24, 25–29, 30–34, 35–39, 40–44, and 45–49 years age group. Ethical approval and informed consent were not required as the GBD data were publicly accessible and the analyses did not involve any identifiable information.

### 2.2 Risk factor

The disease burden of MSMIs attributable to iron deficiency was analyzed based on the comparative risk assessment framework in GBD 2021, which was the mainly contributor to MSMIs-related deaths and DALYs. This study assessed the impact of iron deficiency on the disease burden of MSMIs from 1990 to 2021.

### 2.3 Statistical analysis

The trends of age-standardized mortality rate (ASMR) and age-standardized DALY rate was calculated using the estimated average percentage change (EAPC), presenting with 95% confidence interval (CI). ASMR and age-standardized DALY rate were presented with 95% uncertainty interval (UI). The ASR per 100,000 persons was calculated using the direct method, which involved multiplying the age-specific rates by the corresponding number of individuals (or weight) in each age group of the reference standard population. The resulting products were then summed and divided by the total weight of the standard population. The EAPC was to describe the change trend of ASRs over a designated time period. The regression equation was: *Y* = α + β*X* + ϵ, where *Y* represented the natural logarithm of ASR, *X* represented the calendar year, α was the intercept term, β denoted the slope or trend, and ϵ was the error term. The EAPC was calculated as: 100 × [exp (β) – 1], indicating the annual percentage change. EAPC > 0: the increase of ASRs. EAPC = 0: the stable of ASRs. EAPC < 0: the decrease of ASRs (13). Pearson correlation coefficient was employed to explore the correlations between ASMR, age-standardized DALY rate and SDI level in different GBD regions. *P* < 0.05 was deemed as significant difference. The Bayesian age-period-cohort (BAPC) model is recognized as an advanced research methodology that surpasses traditional analyses in health and socio-economic development studies. The BAPC model facilitates the determination of net drift and local drift, representing overall time trends and specific time trends, while also estimating the impact of the fundamental time dimensions: age, period, and cohort. The future burden tendency of MSMIs attributable to iron deficiency was projected using the BAPC model in the next 30 years. All statistical analyses and visualizations were performed using R software (version 4.4.1).

## 3 Results

### 3.1 Global trends of MSMIs attributable to iron deficiency

Globally, the number of MSMIs-related deaths attributable to iron deficiency declined from 1990 to 2021 by 1.41 times, with a total of 4,420 cases (95% UI: 2,181–5,967) to 3,145 cases (95% UI: 1,515–4,518). And the ASMR decreased from 0.33 per 100,000 persons (95% UI: 0.16–0.45) in 1990 to 0.16 per 100,000 persons (95% UI: 0.08–0.23) in 2021 with an EAPC of −2.49 (95% CI: −2.91 to −2.06; Table 1). Similarly, the downward trend of age-standardized DALY rate was observed from 21.60 per 100,000 persons (95% UI: 10.51–29.05) in 1990 to 10.45 per 100,000 persons (95% UI: 4.96–14.87) in 2021, with an EAPC of −2.51 (95% CI: −2.92 to −2.10; Table 1). These findings indicated that the global burdens of deaths and DALYs in MSMIs attributable to iron deficiency were decreased in the past 30 years.

**Table 1:** Global and regional trends in the burden of MSMIs attributable to iron deficiency: deaths and disability-adjusted life years (1990–2021).

**Location name**	**1990**		**2021**		**EAPC (95% CI)**
	**Number**	**ASR**	**Number**	**ASR**	
**Deaths**					
Global	4420 (2181–5967)	0.33 (0.16–0.45)	3145 (1515–4518)	0.16 (0.08–0.23)	−2.49 (−2.91 to −2.06)
**SDI regions**					
High SDI	18 (8–27)	0.01 (0–0.01)	3 (1–5)	0 (0–0)	−4.45 (−5.07 to −3.83)
High-middle SDI	118 (53–176)	0.04 (0.02–0.06)	16 (7–23)	0.01 (0–0.01)	−6.26 (−6.45 to −6.06)
Middle SDI	649 (281–923)	0.15 (0.06–0.21)	261 (121–372)	0.04 (0.02–0.06)	−4.13 (−4.50 to −3.76)
Low-middle SDI	1465 (738–1964)	0.54 (0.27–0.72)	711 (321–987)	0.14 (0.06–0.19)	−4.48 (−5.14 to −3.82)
Low SDI	2166 (1070–2901)	1.94 (0.96–2.60)	2151 (1044–3187)	0.78 (0.38–1.16)	−3.06 (−3.51 to −2.62)
**Continents**					
Africa	2553 (1233–3499)	1.75 (0.85–2.40)	2187 (1049–3227)	0.63 (0.30–0.94)	−3.25 (−3.56 to −2.94)
America	259 (120–361)	0.14 (0.06–0.19)	112 (52–165)	0.04 (0.02–0.06)	−3.54 (−3.81 to −3.28)
Asia	1553 (769–2092)	0.19 (0.10–0.26)	839 (383–1185)	0.07 (0.03–0.10)	−3.55 (−4.32 to −2.78)
Europe	51 (21–78)	0.03 (0.01–0.04)	4 (2–7)	0 (0–0)	−7.59 (−7.90 to −7.28)
**WHO regions**					
African Region	2225 (1052–3042)	1.91 (0.90–2.61)	1988 (946–2919)	0.69 (0.33–1.01)	−3.25 (−3.60 to −2.90)
Eastern Mediterranean Region	672 (323–959)	0.81 (0.39–1.15)	397 (184–630)	0.21 (0.10–0.33)	−4.68 (−4.84 to −4.51)
European Region	57 (24–85)	0.03 (0.01–0.04)	7 (3–11)	0 (0–0.01)	−6.15 (−6.43 to −5.87)
Region of the Americas	259 (120–361)	0.14 (0.06–0.19)	112 (52–165)	0.04 (0.02–0.06)	−3.54 (−3.81 to −3.28)
South-East Asia Region	1009 (507–1360)	0.32 (0.16–0.43)	604 (274–863)	0.11 (0.05–0.16)	−3.84 (−4.86 to −2.81)
Western Pacific Region	194 (83–291)	0.05 (0.02–0.07)	33 (15–51)	0.01 (0–0.01)	−6.12 (−6.51 to −5.73)
**Global burden of disease regions**					
East Asia	125 (50–201)	0.04 (0.01–0.06)	4 (2–7)	0 (0–0)	−10.55 (−10.92 to −10.18)
Oceania	6 (2–11)	0.40 (0.15–0.70)	15 (7–24)	0.42 (0.20–0.68)	0.38 (0.24 to 0.53)
Southeast Asia	298 (129–430)	0.25 (0.11–0.36)	63 (28–96)	0.03 (0.02–0.05)	−6.38 (−6.56 to −6.19)
Central Sub-Saharan Africa	255 (116–384)	2.06 (0.94–3.11)	429 (177–732)	1.31 (0.54–2.24)	−0.80 (−1.51 to −0.08)
Eastern Sub-Saharan Africa	961 (451–1351)	2.23 (1.04–3.13)	596 (279–915)	0.56 (0.26–0.85)	−4.79 (−5.17 to −4.41)
Southern Sub-Saharan Africa	97 (44–148)	0.73 (0.33–1.12)	45 (19–73)	0.21 (0.09–0.34)	−2.43 (−3.51 to −1.33)
Western Sub-Saharan Africa	974 (453–1404)	2.23 (1.04–3.22)	1059 (513–1621)	0.88 (0.43–1.35)	−3.17 (−3.45 to −2.89)
South Asia	1000 (520–1380)	0.39 (0.20–0.54)	658 (305–936)	0.13 (0.06–0.19)	−4.02 (−5.04 to −2.99)
Andean Latin America	60 (28–88)	0.63 (0.29–0.93)	17 (7–28)	0.10 (0.04–0.16)	−6.73 (−7.27 to −6.19)
Caribbean	39 (18–57)	0.42 (0.19–0.61)	48 (23–80)	0.40 (0.19–0.67)	0.18 (−0.09 to 0.46)
Central Latin America	72 (31–105)	0.17 (0.07–0.25)	20 (9–31)	0.03 (0.01–0.05)	−5.62 (−6.07 to −5.17)
Tropical Latin America	66 (32–94)	0.17 (0.08–0.24)	22 (10–33)	0.04 (0.02–0.05)	−4.05 (−4.39 to −3.71)
North Africa and Middle East	405 (189–566)	0.52 (0.24–0.72)	157 (68–258)	0.10 (0.04–0.16)	−5.31 (−5.42 to −5.2)
Central Asia	9 (4–12)	0.05 (0.02–0.07)	4 (2–6)	0.02 (0.01–0.02)	−3.52 (−3.73 to −3.31)
Central Europe	7 (3–11)	0.02 (0.01–0.04)	0 (0–0)	0 (0–0)	−9.59 (−9.94 to −9.24)
Eastern Europe	13 (5–19)	0.02 (0.01–0.03)	1 (0–2)	0 (0–0)	−6.96 (−7.3 to −6.62)
Australasia	0 (0–0)	0 (0–0)	0 (0–0)	0 (0–0)	−6.09 (−6.79 to −5.38)
High-income Asia Pacific	4 (2–6)	0.01 (0–0.01)	0 (0–0)	0 (0–0)	−8.27 (−9.15 to −7.38)
High-income North America	2 (1–4)	0 (0–0)	2 (1–3)	0 (0–0)	−0.42 (−0.87 to 0.03)
Southern Latin America	21 (9–31)	0.17 (0.07–0.25)	4 (2–7)	0.03 (0.01–0.04)	−4.88 (−5.32 to −4.44)
Western Europe	5 (2–8)	0.01 (0–0.01)	1 (0–1)	0 (0–0)	−5.62 (−6.08 to −5.16)
**Disability-adjusted life years**					
Global	288874 (140577–388518)	21.60 (10.51–29.05)	203691 (96593–289761)	10.45 (4.96–14.87)	−2.51 (−2.92 to −2.10)
**SDI regions**					
High SDI	2628 (1029–4588)	1.16 (0.45–2.02)	1141 (369–2196)	0.47 (0.15–0.90)	−2.64 (−2.88 to −2.40)
High-middle SDI	9764 (4099–14857)	3.52 (1.48–5.35)	2326 (895–4094)	0.76 (0.29–1.34)	−4.42 (−4.68 to −4.15)
Middle SDI	45926 (20638–65289)	10.27 (4.62–14.6)	19227 (8534–27443)	3.11 (1.38–4.44)	−3.94 (−4.24 to −3.63)
Low-middle SDI	97144 (48474–130624)	35.59 (17.76–47.86)	47444 (21551–66307)	9.37 (4.26–13.1)	−4.48 (−5.11 to −3.85)
Low SDI	133203 (66321–177847)	119.27 (59.38–159.24)	133363 (64300–197371)	48.62 (23.44–71.95)	−3.05 (−3.49 to −2.60)
**Continents**					
Africa	156779 (76115–214282)	107.71 (52.29–147.22)	136079 (64546–199876)	39.47 (18.72–57.97)	−3.21 (−3.51 to −2.91)
America	18292 (8201–25855)	9.83 (4.41–13.90)	8691 (3704–12985)	3.36 (1.43–5.02)	−3.23 (−3.46 to −3)
Asia	108796 (53129–147949)	13.51 (6.6–18.37)	57495 (26067–80263)	4.99 (2.26–6.96)	−3.61 (−4.33 to −2.89)
Europe	4770 (1843–7525)	2.41 (0.93–3.80)	1229 (411–2359)	0.65 (0.22–1.24)	−3.71 (−4.01 to −3.42)
**WHO regions**					
African Region	136499 (64717–186353)	116.95 (55.45–159.67)	123884 (58435–182035)	43.07 (20.31–63.28)	−3.20 (−3.55 to −2.86)
Eastern Mediterranean Region	42602 (20600–59804)	51.04 (24.68–71.64)	25513 (11798–39564)	13.28 (6.14–20.60)	−4.61 (−4.77 to −4.45)
European Region	5276 (2075–8246)	2.49 (0.98–3.90)	1569 (574–2890)	0.74 (0.27–1.36)	−3.35 (−3.63 to −3.08)
Region of the Americas	18292 (8201–25855)	9.83 (4.41–13.90)	8691 (3704–12985)	3.36 (1.43–5.02)	−3.23 (−3.46 to −3)
South-East Asia Region	70457 (35100–96443)	22.29 (11.11–30.51)	40325 (18449–56968)	7.29 (3.34–10.30)	−4.01 (−4.98 to −3.02)
Western Pacific Region	15475 (6664–23480)	3.72 (1.60–5.65)	3500 (1409–5728)	0.80 (0.32–1.31)	−4.90 (−5.29 to −4.51)
**Global burden of disease regions**					
East Asia	10631 (4181–16973)	3.19 (1.25–5.09)	1207 (395–2410)	0.36 (0.12–0.73)	−6.55 (−7.14 to −5.96)
Oceania	414 (161–705)	26.63 (10.37–45.35)	885 (414–1417)	25.50 (11.92–40.82)	0.09 (−0.04 to 0.23)
Southeast Asia	19609 (8490–28197)	16.32 (7.06–23.46)	4905 (2174–7560)	2.68 (1.19–4.13)	−5.82 (−5.96 to −5.69)
Central Sub-Saharan Africa	15520 (7014–23389)	125.55 (56.74–189.21)	25777 (10670–44247)	78.94 (32.68–135.51)	−0.85 (−1.54 to −0.14)
Eastern Sub-Saharan Africa	57642 (27255–80506)	133.58 (63.16–186.57)	36830 (17206–56133)	34.39 (16.07–52.41)	−4.67 (−5.04 to −4.29)
Southern Sub-Saharan Africa	6183 (2780–9452)	46.52 (20.91–71.11)	2918 (1266–4697)	13.44 (5.83–21.63)	−2.41 (−3.45 to −1.35)
Western Sub-Saharan Africa	60508 (28268–87356)	138.76 (64.83–200.34)	66620 (32002–102104)	55.57 (26.69–85.16)	−3.14 (−3.42 to −2.86)
South Asia	69663 (35720–96138)	27.33 (14.01–37.72)	43845 (20070–62648)	8.87 (4.06–12.68)	−4.17 (−5.14 to −3.19)
Andean Latin America	3852 (1773–5561)	40.61 (18.69–58.63)	1233 (535–2007)	7.06 (3.06–11.50)	−6.21 (−6.71 to −5.71)
Caribbean	2465 (1133–3605)	26.45 (12.15–38.67)	2909 (1366–4732)	24.18 (11.36–39.34)	0.03 (−0.23 to 0.30)
Central Latin America	5179 (2169–7759)	12.36 (5.18–18.51)	1813 (752–2914)	2.66 (1.1–4.27)	−4.84 (−5.22 to −4.47)
Tropical Latin America	4650 (2183–6566)	11.66 (5.47–16.46)	1908 (836–2836)	3.15 (1.38–4.68)	−3.38 (−3.70 to −3.07)
North Africa and Middle East	26297 (12129–36776)	33.66 (15.53–47.07)	10438 (4502–16886)	6.55 (2.83–10.60)	−5.22 (−5.32 to −5.12)
Central Asia	709 (323–1026)	4.22 (1.92–6.12)	381 (181–606)	1.57 (0.74–2.50)	−2.82 (−3.04 to −2.60)
Central Europe	689 (263–1127)	2.24 (0.86–3.67)	120 (40–237)	0.47 (0.15–0.92)	−4.54 (−4.92 to −4.16)
Eastern Europe	1331 (507–2231)	2.41 (0.92–4.04)	427 (139–831)	0.89 (0.29–1.72)	−2.35 (−2.72 to −1.98)
Australasia	29 (9–62)	0.54 (0.17–1.15)	34 (9–73)	0.47 (0.13–1.02)	0.11 (−0.19 to 0.41)
High-income Asia Pacific	368 (152–615)	0.81 (0.33–1.34)	81 (26–162)	0.21 (0.07–0.43)	−4.13 (−4.59 to −3.66)
High-income North America	779 (271–1508)	1.05 (0.36–2.03)	492 (159–936)	0.59 (0.19–1.11)	−2.16 (−2.27 to −2.05)
Southern Latin America	1479 (656–2190)	11.94 (5.3–17.67)	440 (166–714)	2.52 (0.95–4.10)	−3.99 (−4.36 to −3.61)
Western Europe	877 (349–1553)	0.92 (0.37–1.63)	427 (131–860)	0.46 (0.14–0.92)	−1.69 (−1.93 to −1.45)

MSMIs, maternal sepsis and other maternal infections; SDI, socio-demographic index; WHO, World Health Organization; ASR, age-standardized rate; EAPC, estimated annual percentage change; CI, confidence interval.

### 3.2 Regional trends of MSMIs attributable to iron deficiency

The global burden of MSMIs attributable to iron deficiency displayed obvious regional differences and was closely associated with SDI levels. From 1990 to 2021, our data reveled that the deaths and DALYs were decreased in all SDI regions. Among them, the decrease of ASMR was the most in the high-middle SDI regions, with an EAPC of −6.26 (95% CI: −6.45 to −6.06), while for age-standardized DALY rate, the decline was the most in the low-middle SDI regions, with an EAPC of −4.48 (95% CI: −5.11 to −3.85; Table 1). Over the study period, the decreases of burden in deaths and DALYs were the most in East Asia, with an EAPC of −10.55 (95% CI: −10.92 to −10.18) and −6.55 (95% CI: −7.14 to −5.96), respectively (Table 1). These results indicated the complex and diverse dynamics of MSMIs attributable to iron deficiency in different regions.

### 3.3 National trends of MSMIs attributable to iron deficiency

**Figure 1:**
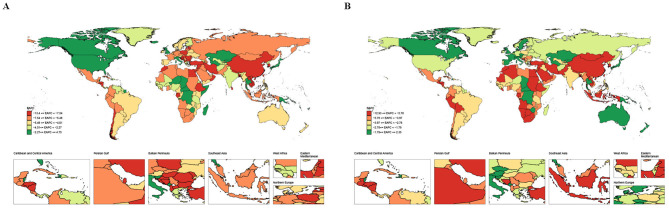
The EAPC map of MSMIs attributable to iron deficiency across 204 countries and territories. **(A)** Deaths. **(B)** DALYs. MSMIs, maternal sepsis and other maternal infections; EAPC, estimated annual percentage change; DALYs, disability-adjusted life years.

The ASMR and age-standardized DALY rate of MSMIs attributable to iron deficiency displayed national variation. In 2021, the highest burdens of MSMIs-related deaths and DALYs attributable to iron deficiency were observed in Somalia, with an ASMR of 2.98 per 100,000 persons (95% UI: 1.30–5.12) and an age-standardized DALY rate of 177.38 per 100,000 persons (95% UI: 76.95–299.71; Figure 1 and Supplementary Table 1). Notably, our data showed that Equatorial Guinea had the largest decrease in ASMR and age-standardized DALY rate, with an EAPC of −13.39 (95% CI: −13.82 to −12.95) and −12.92 (95% CI: −13.34 to −12.49), respectively (Figure 1 and Supplementary Table 1). The significant variation in deaths and DALYs across countries suggested disparities in the healthcare systems and prevention strategies for MSMIs attributable to iron deficiency.

### 3.4 Fractions of MSMIs attributable to iron deficiency in different regions

**Figure 2:**
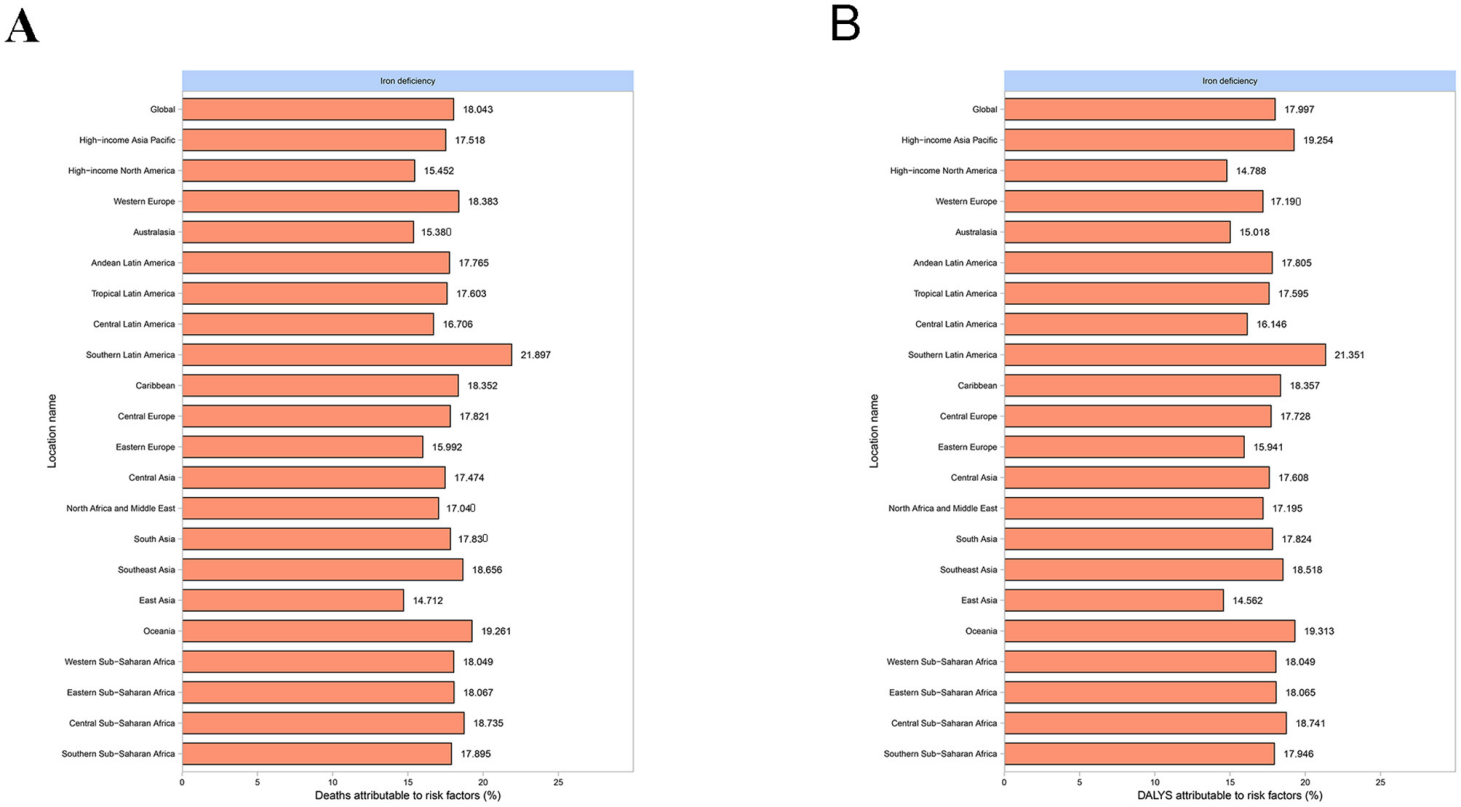
Fraction of MSMIs attributable to iron deficiency in global and GBD regions, 2021. **(A)** Deaths. **(B)** DALYs. MSMIs, maternal sepsis and other maternal infections; GBD, global burden of disease; DALYs, disability-adjusted life years.

**Figure 3:**
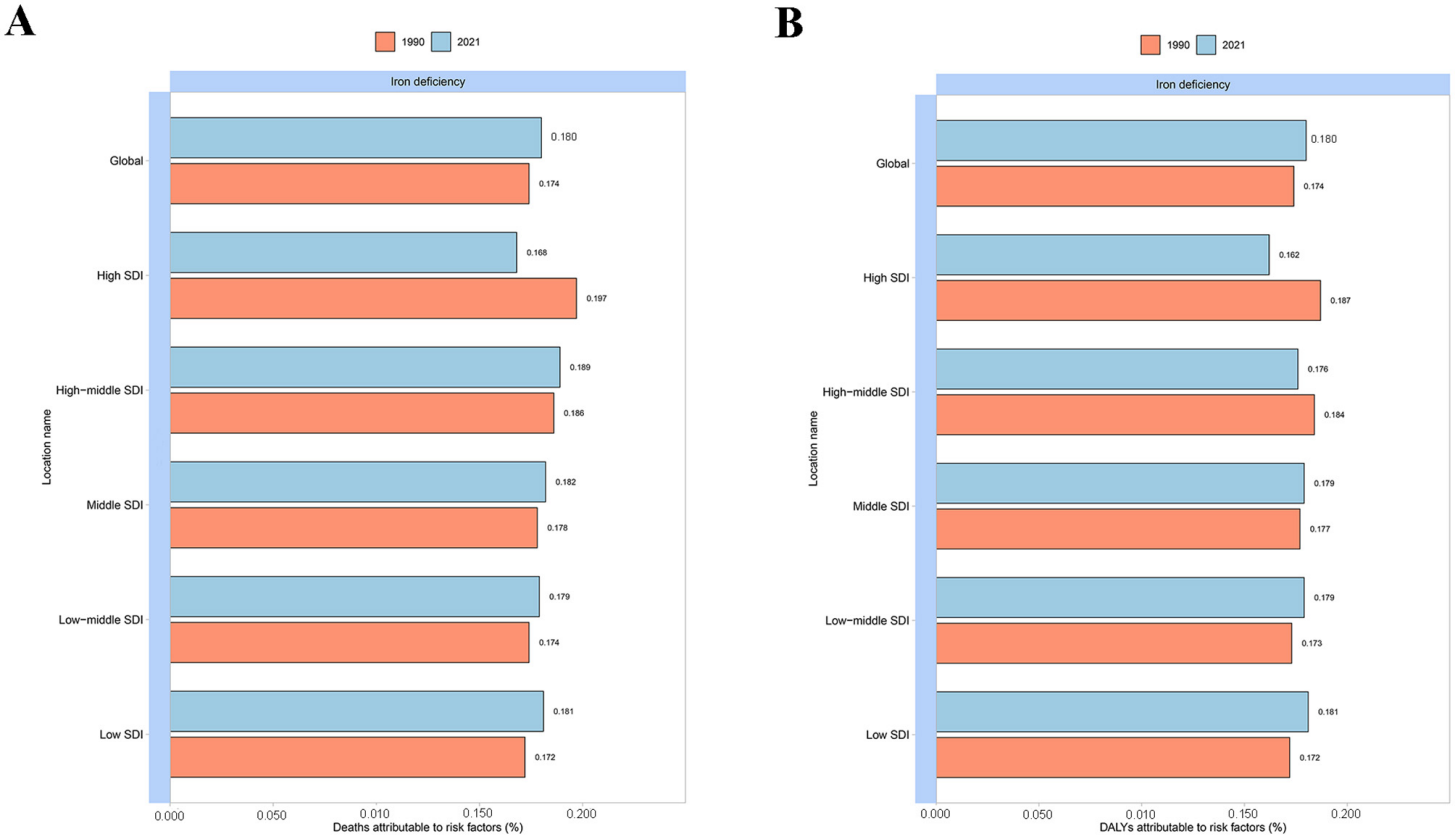
Fraction of MSMIs attributable to iron deficiency across global and SDI regions in 1990 and 2021. **(A)** Deaths. **(B)** DALYs. MSMIs, maternal sepsis and other maternal infections; SDI, socio-demographic index; DALYs, disability-adjusted life years.

In 2021, the global fractions of MSMIs-related deaths and DALYs attributable to iron deficiency were 18.043 and 17.997%, respectively (Figure 2). In all GBD regions, the fractions of deaths (21.897%) and DALYs (21.351%) attributable to iron deficiency were the highest in Southern Latin America (Figure 2). Moreover, the global fractions of MSMIs-related deaths and DALYs attributable to iron deficiency were more in 2021 than 1990 (0.180 vs. 0.174%, Figure 3). In the high SDI regions, it was observed that the fractions of MSMIs-related deaths (0.168%) and DALYs (0.162%) attributable to iron deficiency were the lowest in 2021 (Figure 3). Low and low-middle SDI regions showed significant increases in attributable fractions over time (e.g., low SDI deaths: +0.009% from 1990 to 2021).

### 3.5 Age patterns

**Figure 4:**
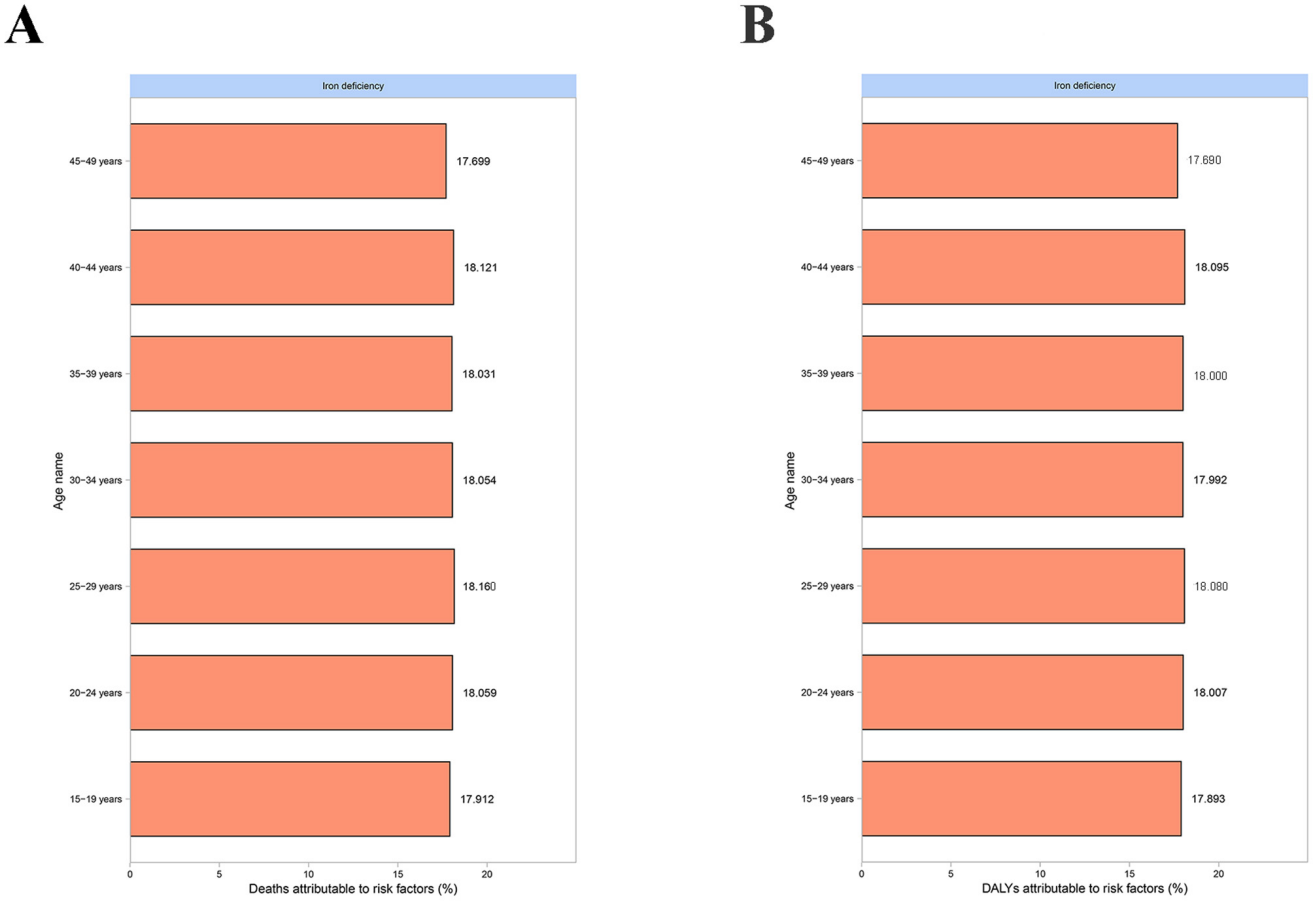
Fraction of MSMIs attributable to iron deficiency in different age subgroups, 2021. **(A)** Deaths. **(B)** DALYs. MSMIs, maternal sepsis and other maternal infections; DALYs, disability-adjusted life years.

**Figure 5:**
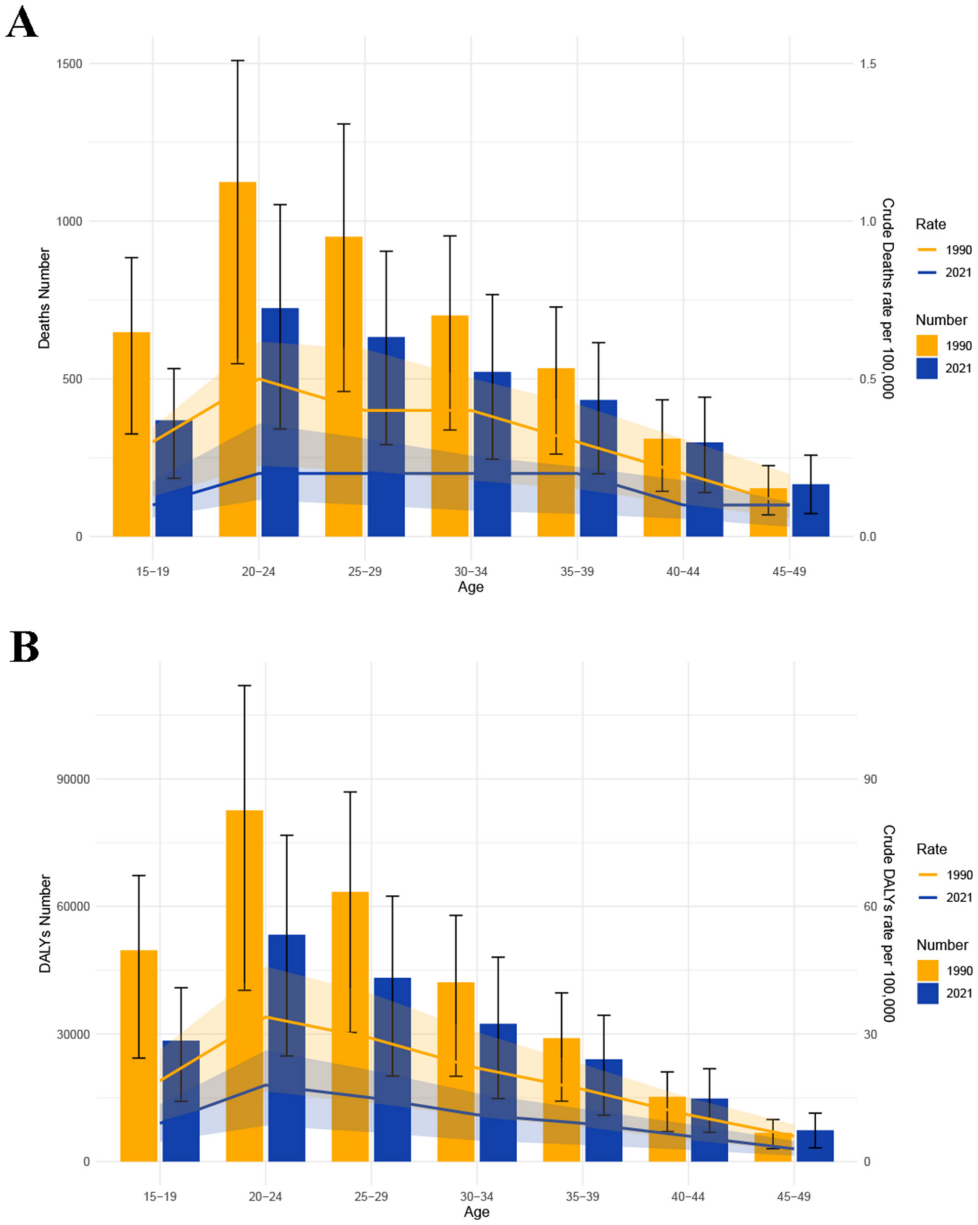
The age-specific numbers of MSMIs attributable to iron deficiency in 1990 and 2021. **(A)** Deaths. **(B)** DALYs. MSMIs, maternal sepsis and other maternal infections; DALYs, disability-adjusted life years.

In 2021, the data showed that the fraction of MSMIs-related deaths attributable to iron deficiency was the most in the 25–29 years age group (18.160%; Figure 4A). Yet, for DALYs, the highest fraction was observed in the 40–44 years age group (18.095%; Figure 4B). Notably, in 1990 and 2021, the peaks of MSMIs-related deaths (1,124 cases and 724 cases) and DALYs (82,593 cases and 53,355 cases) were found in the 20–24 years age group (Figure 5). In MSMIs patients aged 25 years and more, the number of deaths and DALYs was declined with age in 1990 and 2021 (Figure 5).

### 3.6 The trends in age-specific rates and ASRs of MSMIs attributable to iron deficiency

**Figure 6:**
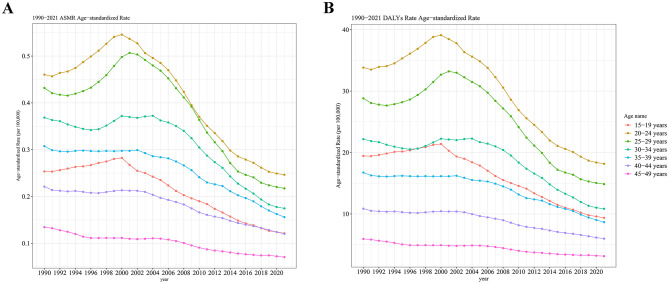
The global trends in age-specific rates of MSMIs attributable to iron deficiency from 1990 to 2021. **(A)** Deaths. **(B)** DALYs. MSMIs, maternal sepsis and other maternal infections; ASMR, age-standardized mortality rate; DALY, disability-adjusted life year.

**Figure 7:**
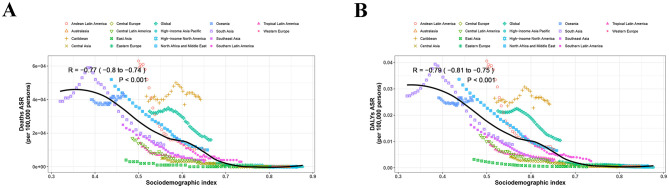
The trend in ASRs of MSMIs attributable to iron deficiency across GBD regions by SDI from 1990 to 2021. **(A)** Deaths. **(B)** DALYs. ASRs, age-standardized rates; MSMIs, maternal sepsis and other maternal infections; GBD, global burden of disease; SDI, socio-demographic index; DALY, disability-adjusted life year.

From 1990 to 2021, it was observed that the ASMR and age-standardized DALY rate were the highest in the 20–24 years age group compared to other age subgroups, and the lowest ASMR and age-standardized DALY rate were found in the 45–49 years group (Figure 6). Since 2000, the burdens of MSMIs-related deaths and DALYs attributable to iron deficiency were continuously declined in the 20–24 years age group (Figure 6). On the other hand, our data revealed that there was a significant negative correlation between the ASMR of MSMIs attributable to iron deficiency and SDI from 1990 to 2021 in different GBD regions (*R* = −0.77 (−0.80 to −0.74), *P* < 0.001, Figure 7A). Similar negative correlation between the age-standardized DALY rate and SDI was observed in different GBD regions (*R* = −0.79 (−0.81 to −0.75), *P* < 0.001, Figure 7B). These findings suggested that the mortality and DALYs were lower in GBD areas with higher SDI.

### 3.7 Future forecast

**Figure 8:**
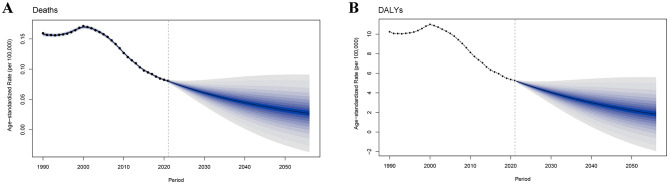
Future forecast of global burden of MSMIs attributable to iron deficiency by 2050. **(A)** Deaths. **(B)** DALYs. MSMIs, maternal sepsis and other maternal infections; DALY, disability-adjusted life year.

The global burden of MSMIs attributable to iron deficiency was predicted from 2021 to 2050 (Figure 8). Our results revealed that the global burden in MSMIs-related deaths attributable to iron deficiency was predicted to decline by 2050, with a decrease of ASMR from 0.08 per 100,000 persons to 0.03 per 100,000 persons (Figure 8A). Similarly, a declined trend in MSMIs-related DALYs attributable to iron deficiency was found, with a decrease of age-standardized DALY rate from 5.27 per 100,000 persons to 2.21 per 100,000 persons (Figure 8B). These findings indicated that the burden of MSMIs attributable to iron deficiency was mitigated in the next 30 years.

## 4 Discussion

As previously described, iron deficiency is a significant risk factor of MSMIs (7, 14, 15). This study utilizes the GBD 2021 database to analyze global trends in the burden of MSMIs attributable to iron deficiency from 1990 to 2021, as well as the contribution of the iron deficiency to the global burden of MSMIs disease.

The specific mechanisms by which iron deficiency increases susceptibility to MSMIs are increasingly understood. Iron is essential for the proliferation, differentiation, and effector functions of immune cells, including neutrophils, macrophages, natural killer (NK) cells, and T lymphocytes (8, 9). Iron deficiency directly impairs neutrophil function, including decreased oxidative burst and bactericidal capacity (9, 16). It also leads to reduced phagocytosis and intracellular killing of pathogens by macrophages (9, 17). In addition, iron deficiency introduces impaired T-cell-mediated immune production, which is essential for defense against common intracellular bacteria associated with maternal infections (9, 18).

In recent years, GBD studies have exhibited differences in the mortality and DALYs of MSMIs across regions and countries, aiding in the identification of high-risk populations and areas (7, 11, 19). The incidence and mortality of maternal sepsis have been reported to exhibit a geographical variation worldwide (20–22). A 2019 study from the United States reveals that 23% of maternal deaths are associated with sepsis, indicating that even high-income countries are not immune to the fatal consequences of maternal infections (23). According to the GBD 2019 study, the global number of MSMIs declines from 23,029,127 cases in 1990 to 20,569,889 cases in 2021 (19). In 2019, the highest incidence and disease burden of MSMIs are observed in the low and low-middle SDI regions (19). Another GBD 2019 study highlights an overall global downward trend in both the incidence and mortality of MSMIs, with substantial regional heterogeneity, indicating the effectiveness and imbalance of global management of MSMIs (11). Moreover, the GBD 2021 study shows a marked reduction in the MSMIs burden in the high SDI regions, while a rise is observed in the low SDI regions, with population growth identified as a major contributing factor (7). The study also reinforces iron deficiency as a key risk factor for MSMIs, particularly in lower SDI regions (7). Based on GBD 2021 data, our study further demonstrates that the global burden of MSMIs-related deaths and DALYs attributable to iron deficiency has shown a declining trend over the past three decades and is projected to decrease further in the next 30 years. This decline may be associated with the implementation of progressive global public health policies. The 2017 Global Maternal Sepsis Study (GLOSS) is initiated to develop and validate criteria for identifying severe maternal infections and sepsis, thereby enabling the implementation of quality improvement strategies for better recognition and management of maternal sepsis (24). This proactive policy has contributed to alleviating the global burden of MSMIs attributable to iron deficiency.

The GBD 2021 report indicates a significantly negative correlation between the SDI levels of different countries and regions and the age-standardized DALY rate for MSMIs (7). In low SDI countries (Somalia, Chad, and the Central African Republic), the burden of MSMIs-related DALYs is the highest, reflecting inadequate healthcare infrastructure and poor socioeconomic conditions. In countries with moderate SDI (Afghanistan, Madagascar, and Zimbabwe), the DALYs burden has improved, but significant disease challenges remain. Conversely, high SDI countries (Western Europe, North America, and East Asia) exhibit the lowest burden of MSMIs-related DALYs, mainly due to advanced healthcare systems and better maternal care (7). The reduction in the disease burden of MSMIs due to iron deficiency in East Asia may be related to the implementation of national policies in recent years that emphasize early iron supplementation before and during pregnancy (25). Of note, an important finding of this study is that in GBD regions with higher SDI levels, MSMIs-related deaths and DALYs attributable to iron deficiency are lower. The differences between SDI regions highlight the need for tailored public health interventions (26, 27). In the high SDI regions, healthcare institutions typically provide high-standard maternal care services, including prenatal check-ups, nutritional guidance, psychological support, and educational programs (28). These measures have effectively reduced the burden of infectious diseases attributable to iron deficiency. In low and low-middle SDI regions, efforts should focus on improving iron intake through fortified foods and supplements, alongside strengthening prenatal care to monitor and manage iron levels. Moreover, public health policies should prioritize education on iron intake during pregnancy and develop infrastructure to support iron supplementation programs.

There is a significant age-related difference in the burden of MSMIs. The GBD 2021 study indicates that pregnant women aged 20–29 years are the most vulnerable, with the incidence and mortality of MSMIs peaking in this age group (7). Our findings show that the burden of MSMIs-related deaths and DALYs due to iron deficiency is highest in the 20–24 years age group. Many women in this age group are beginning or are in the reproductive stage of their lives. Although overall health may be better in this age group, iron deficiency is often overlooked or not addressed in a timely manner. During pregnancy, the demand for iron increases significantly, and if iron supplementation is delayed, it may lead to iron deficiency anemia (29–31). Anemia increases the risk of maternal infections, particularly life-threatening infectious sepsis disease (32, 33). In Tanzania, pregnant women aged 20–24 years are at a higher risk of iron deficiency (34). This may be attributed to irregular diets, unhealthy weight loss practices, and insufficient knowledge of nutrition, which prevent many young women from effectively consuming adequate iron (35, 36). In impoverished regions, women in the 20–24 years age group may lack sufficient health education and resources, making them unaware of the dangers of iron deficiency and failing to receive proper nutritional supplementation, further increasing the risk of pregnancy-related infections (37). The highest absolute burden of iron deficiency attributable to MSMIs was observed among the 20–24 age group. However, the attributable fraction of iron deficiency peaked in older age groups, with the highest rates observed among 25–29 years for deaths and 40–44 years for DALYs. This apparent discrepancy can be attributed to these factors: (1) Higher prevalence of chronic iron deficiency in older pregnant women due to prolonged iron depletion from repeated pregnancies and age-related reduced absorption; (2) Increase in disability weights with age; and (3) Larger population of younger women, leading to higher absolute case numbers even if their relative risk from iron deficiency is lower than in older groups. Moreover, our findings show that after 2000, the burden of MSMIs-related deaths and DALYs due to iron deficiency in the 20–24 years age group has shown a consistent decline. As early as 2000, the United States government began encouraging pregnant women to take iron supplements during pregnancy and provided education on iron intake to ensure they received adequate iron and reduced the risk of infections due to anemia (38). This proactive measure has effectively reduced maternal anemia, helping alleviate the global burden of MSMIs attributable to iron deficiency.

In present study, a comprehensive assessment of the global burden of MSMIs attributable to iron deficiency has been conducted. Yet, several limitations should be acknowledged. Firstly, the GBD 2021 database does not record the prevalence and incidence of MSMIs attributable to iron deficiency, which may compromise the accuracy of epidemiological trend assessments for this condition. Secondly, the GBD database does not differentiate between specific sources of maternal infections, instead focusing on the clinical manifestations of MSMIs. This may obscure important distinctions in etiology and outcomes. Thirdly, due to regional disparities in the medical diagnostic capabilities for MSMIs attributable to iron deficiency worldwide, the actual burden of MSMIs attributable to iron deficiency may be underestimated.

## 5 Conclusions

To sum up, the global burden of MSMIs attributable to iron deficiency has shown an overall downward trend from 1990 to 2021, which is predicted to continually decline in the next 30 years. The greatest decrease in global burden for MSMIs-related deaths attributable to iron deficiency is in the high-middle SDI regions, while for DALYs, the greatest decrease is in the low-middle SDI regions. The peaks of MSMIs-related deaths and DALYs were found in the 20–24 years age group. Moreover, the ASRs of deaths and DALYs are negatively correlated with SDI from 1990 to 2021 in different GBD regions. These findings will provide critical support for mitigating the global burden of MSMIs attributable to iron deficiency. Future large-scale cohort studies are needed to validate and support these findings.

## Data Availability

Publicly available datasets were analyzed in this study. This data can be found here: https://ghdx.healthdata.org.
