# Supplementary material for: Trends in the disease burden of maternal sepsis and other maternal infections attributable to iron deficiency from 1990 to 2021 and its projection until 2050
Source: Front Public Health. 2025 Sep 4;13:1658505. doi: 10.3389/fpubh.2025.1658505 (PMC12443840; doi:10.3389/fpubh.2025.1658505)
Supplement: Supplementary file 1 [file Table_1.docx]

**Supplementary Table 1** The global burden of MSMIs attributable to iron deficiency in 204 countries and territories.

| Location name | 1990 | | 2021 | | EAPC (95% CI) |
| --- | --- | --- | --- | --- | --- |
|  | Number | ASR | Number | ASR |  |
| Deaths |  |  |  |  |  |
| Afghanistan | 28 (11-48) | 1.27 (0.48-2.19) | 53 (19-102) | 0.73 (0.26-1.42) | -2.28 (-3.05 to -1.51) |
| Albania | 0 (0-0) | 0.03 (0.01-0.05) | 0 (0-0) | 0 (0-0) | -8.98 (-9.55 to -8.42) |
| Algeria | 32 (13-51) | 0.55 (0.23-0.87) | 6 (2-12) | 0.06 (0.02-0.10) | -7.21 (-7.37 to -7.04) |
| American Samoa | 0 (0-0) | 0.06 (0.02-0.10) | 0 (0-0) | 0.09 (0.04-0.17) | 1.85 (1.47 to 2.24) |
| Andorra | 0 (0-0) | 0 (0-0) | 0 (0-0) | 0 (0-0) | -4.45 (-4.54 to -4.36) |
| Angola | 53 (22-89) | 2.29 (0.94-3.87) | 30 (12-54) | 0.39 (0.16-0.70) | -5.89 (-6.15 to -5.64) |
| Antigua and Barbuda | 0 (0-0) | 0.10 (0.04-0.15) | 0 (0-0) | 0.03 (0.01-0.04) | -2.69 (-3.48 to -1.89) |
| Argentina | 16 (7-24) | 0.20 (0.09-0.30) | 4 (2-6) | 0.03 (0.01-0.05) | -4.56 (-4.95 to -4.16) |
| Armenia | 0 (0-0) | 0.03 (0.01-0.05) | 0 (0-0) | 0.01 (0-0.01) | -4.78 (-5.48 to -4.06) |
| Australia | 0 (0-0) | 0 (0-0) | 0 (0-0) | 0 (0-0) | -4.81 (-5.42 to -4.2) |
| Austria | 0 (0-0) | 0 (0-0) | 0 (0-0) | 0 (0-0) | -5.26 (-5.79 to -4.73) |
| Azerbaijan | 0 (0-0) | 0.01 (0-0.01) | 0 (0-0) | 0 (0-0) | -7.12 (-7.46 to -6.79) |
| Bahamas | 0 (0-0) | 0.04 (0.02-0.06) | 0 (0-0) | 0.01 (0.01-0.02) | -2.46 (-3.36 to -1.55) |
| Bahrain | 0 (0-0) | 0.03 (0.02-0.06) | 0 (0-0) | 0.01 (0-0.01) | -4.82 (-5.23 to -4.41) |
| Bangladesh | 54 (26-76) | 0.22 (0.10-0.31) | 2 (1-3) | 0 (0-0.01) | -12.5 (-13.36 to -11.63) |
| Barbados | 0 (0-0) | 0.10 (0.04-0.14) | 0 (0-0) | 0.04 (0.02-0.06) | -2.55 (-3.34 to -1.76) |
| Belarus | 0 (0-1) | 0.02 (0.01-0.03) | 0 (0-0) | 0 (0-0) | -5.47 (-5.97 to -4.97) |
| Belgium | 0 (0-0) | 0 (0-0) | 0 (0-0) | 0 (0-0) | -5.27 (-5.85 to -4.68) |
| Belize | 0 (0-0) | 0.14 (0.06-0.20) | 0 (0-0) | 0.06 (0.03-0.10) | -0.93 (-1.54 to -0.32) |
| Benin | 21 (9-33) | 1.96 (0.87-3.04) | 11 (5-18) | 0.33 (0.14-0.55) | -5.38 (-5.79 to -4.97) |
| Bermuda | 0 (0-0) | 0.01 (0-0.02) | 0 (0-0) | 0 (0-0) | -9.24 (-10.83 to -7.62) |
| Bhutan | 1 (0-1) | 0.36 (0.16-0.61) | 0 (0-0) | 0.02 (0.01-0.03) | -9.81 (-9.96 to -9.66) |
| Bolivia (Plurinational State of) | 17 (7-27) | 1.12 (0.47-1.79) | 5 (2-10) | 0.17 (0.07-0.31) | -6.36 (-6.59 to -6.14) |
| Bosnia and Herzegovina | 0 (0-1) | 0.04 (0.01-0.06) | 0 (0-0) | 0 (0-0) | -11.19 (-12.32 to -10.05) |
| Botswana | 4 (2-7) | 1.30 (0.54-2.32) | 1 (0-1) | 0.10 (0.04-0.17) | -7.36 (-7.94 to -6.78) |
| Brazil | 63 (30-88) | 0.16 (0.08-0.23) | 21 (9-30) | 0.03 (0.02-0.05) | -4.07 (-4.45 to -3.7) |
| Brunei Darussalam | 0 (0-0) | 0.01 (0-0.01) | 0 (0-0) | 0 (0-0) | -4.72 (-5.22 to -4.22) |
| Bulgaria | 0 (0-1) | 0.02 (0.01-0.04) | 0 (0-0) | 0 (0-0) | -7.95 (-8.40 to -7.50) |
| Burkina Faso | 35 (14-55) | 1.66 (0.66-2.63) | 36 (17-59) | 0.66 (0.30-1.08) | -3.62 (-3.94 to -3.30) |
| Burundi | 42 (17-72) | 3.31 (1.34-5.71) | 34 (13-60) | 1.1 (0.42-1.92) | -4.39 (-4.81 to -3.96) |
| Cabo Verde | 0 (0-0) | 0.37 (0.15-0.63) | 0 (0-0) | 0.01 (0-0.02) | -11.46 (-11.75 to -11.17) |
| Cambodia | 17 (8-28) | 0.70 (0.32-1.10) | 4 (1-6) | 0.08 (0.03-0.14) | -8.51 (-9.24 to -7.77) |
| Cameroon | 22 (9-35) | 0.91 (0.37-1.46) | 14 (6-24) | 0.18 (0.08-0.31) | -4.57 (-5.17 to -3.96) |
| Canada | 0 (0-0) | 0 (0-0.01) | 0 (0-0) | 0 (0-0.01) | 0.12 (-0.30 to 0.55) |
| Central African Republic | 18 (8-30) | 2.87 (1.18-4.70) | 27 (10-49) | 1.96 (0.73-3.57) | -1.18 (-1.43 to -0.92) |
| Chad | 54 (25-82) | 4.09 (1.86-6.18) | 103 (49-169) | 2.66 (1.26-4.38) | -1.58 (-1.94 to -1.21) |
| Chile | 4 (2-6) | 0.11 (0.05-0.18) | 0 (0-1) | 0.01 (0-0.01) | -8.38 (-9.23 to -7.52) |
| China | 124 (50-200) | 0.04 (0.02-0.06) | 4 (1-7) | 0 (0-0) | -10.64 (-11.02 to -10.25) |
| Colombia | 8 (3-13) | 0.09 (0.04-0.15) | 2 (1-3) | 0.01 (0-0.02) | -6.46 (-7.08 to -5.83) |
| Comoros | 1 (0-2) | 0.85 (0.3-1.44) | 0 (0-1) | 0.20 (0.08-0.39) | -5.54 (-6.05 to -5.02) |
| Congo | 7 (3-12) | 1.28 (0.54-2.10) | 5 (2-9) | 0.35 (0.15-0.62) | -4.07 (-4.64 to -3.50) |
| Cook Islands | 0 (0-0) | 0 (0-0) | 0 (0-0) | 0 (0-0) | -1.52 (-2.67 to -0.36) |
| Costa Rica | 1 (0-1) | 0.11 (0.05-0.17) | 0 (0-0) | 0.01 (0.01-0.02) | -6.87 (-7.15 to -6.58) |
| Croatia | 0 (0-0) | 0 (0-0.01) | 0 (0-0) | 0 (0-0) | -8.80 (-9.58 to -8.02) |
| Cuba | 2 (1-3) | 0.07 (0.03-0.10) | 0 (0-0) | 0.01 (0-0.02) | -5.63 (-6.30 to -4.94) |
| Cyprus | 0 (0-0) | 0 (0-0) | 0 (0-0) | 0 (0-0) | -4.04 (-4.29 to -3.79) |
| Czechia | 0 (0-0) | 0 (0-0) | 0 (0-0) | 0 (0-0) | -6.80 (-7.63 to -5.96) |
| Democratic People's Republic of Korea | 1 (0-2) | 0.01 (0.01-0.03) | 0 (0-1) | 0.01 (0-0.01) | -2.72 (-3.20 to -2.23) |
| Democratic Republic of the Congo | 171 (75-266) | 2.00 (0.88-3.12) | 366 (149-631) | 1.72 (0.70-2.96) | 0.32 (-0.58 to 1.22) |
| Denmark | 0 (0-0) | 0 (0-0) | 0 (0-0) | 0 (0-0) | -5.12 (-5.62 to -4.61) |
| Djibouti | 2 (1-3) | 1.60 (0.71-2.65) | 2 (1-3) | 0.50 (0.18-0.94) | -3.76 (-4.09 to -3.44) |
| Dominica | 0 (0-0) | 0.15 (0.07-0.24) | 0 (0-0) | 0.08 (0.03-0.14) | -1.51 (-2.31 to -0.7) |
| Dominican Republic | 3 (1-4) | 0.14 (0.06-0.21) | 1 (0-2) | 0.03 (0.01-0.06) | -3.4 (-4.04 to -2.75) |
| Ecuador | 8 (3-13) | 0.31 (0.13-0.50) | 2 (1-3) | 0.04 (0.01-0.06) | -5.73 (-6.49 to -4.96) |
| Egypt | 56 (24-91) | 0.43 (0.18-0.69) | 6 (2-10) | 0.02 (0.01-0.04) | -8.30 (-8.89 to -7.71) |
| El Salvador | 6 (2-9) | 0.42 (0.15-0.71) | 0 (0-0) | 0.01 (0-0.02) | -10.31 (-12.10 to -8.49) |
| Equatorial Guinea | 4 (2-7) | 4.11 (1.73-6.97) | 0 (0-1) | 0.08 (0.03-0.17) | -13.39 (-13.82 to -12.95) |
| Eritrea | 41 (19-61) | 5.25 (2.45-7.74) | 22 (9-41) | 1.35 (0.54-2.46) | -3.92 (-4.23 to -3.61) |
| Estonia | 0 (0-0) | 0.02 (0.01-0.03) | 0 (0-0) | 0 (0-0) | -9.94 (-10.19 to -9.68) |
| Eswatini | 1 (0-2) | 0.45 (0.17-0.78) | 0 (0-1) | 0.13 (0.04-0.27) | -3.08 (-3.65 to -2.50) |
| Ethiopia | 344 (140-516) | 3.05 (1.24-4.57) | 77 (31-133) | 0.28 (0.11-0.48) | -8.61 (-9.25 to -7.95) |
| Fiji | 0 (0-0) | 0.07 (0.03-0.13) | 0 (0-0) | 0.04 (0.02-0.08) | -1.44 (-1.75 to -1.13) |
| Finland | 0 (0-0) | 0.01 (0-0.01) | 0 (0-0) | 0 (0-0) | -3.86 (-4.33 to -3.39) |
| France | 0 (0-0) | 0 (0-0) | 0 (0-0) | 0 (0-0) | -5.71 (-6.04 to -5.38) |
| Gabon | 2 (1-2) | 0.72 (0.33-1.09) | 0 (0-1) | 0.06 (0.02-0.11) | -8.17 (-8.59 to -7.74) |
| Gambia | 5 (2-8) | 2.09 (0.97-3.37) | 4 (2-7) | 0.65 (0.28-1.11) | -3.73 (-4.02 to -3.44) |
| Georgia | 1 (0-1) | 0.04 (0.02-0.06) | 0 (0-0) | 0.01 (0.01-0.02) | -2.33 (-2.95 to -1.7) |
| Germany | 4 (2-6) | 0.02 (0.01-0.03) | 0 (0-0) | 0 (0-0) | -6.89 (-7.55 to -6.23) |
| Ghana | 40 (17-67) | 1.13 (0.47-1.90) | 16 (7-26) | 0.17 (0.08-0.29) | -5.89 (-6.05 to -5.73) |
| Greece | 0 (0-0) | 0 (0-0.01) | 0 (0-0) | 0 (0-0) | -3.09 (-3.72 to -2.46) |
| Greenland | 0 (0-0) | 0 (0-0) | 0 (0-0) | 0 (0-0) | -2.87 (-3.13 to -2.61) |
| Grenada | 0 (0-0) | 0.01 (0-0.01) | 0 (0-0) | 0 (0-0) | -2.48 (-3.28 to -1.68) |
| Guam | 0 (0-0) | 0.01 (0-0.02) | 0 (0-0) | 0 (0-0.01) | -2.51 (-3.27 to -1.75) |
| Guatemala | 23 (10-33) | 1.24 (0.56-1.78) | 8 (3-12) | 0.18 (0.08-0.27) | -6.38 (-7.17 to -5.59) |
| Guinea | 45 (21-68) | 3.31 (1.54-5.00) | 32 (13-57) | 0.97 (0.40-1.70) | -3.64 (-3.78 to -3.50) |
| Guinea-Bissau | 3 (1-6) | 1.43 (0.63-2.41) | 2 (1-4) | 0.44 (0.20-0.80) | -3.36 (-3.57 to -3.15) |
| Guyana | 0 (0-0) | 0.11 (0.05-0.17) | 0 (0-0) | 0.05 (0.02-0.08) | -2.35 (-2.68 to -2.01) |
| Haiti | 32 (15-49) | 2.05 (0.94-3.17) | 45 (21-75) | 1.27 (0.59-2.13) | -1.30 (-1.61 to -1.00) |
| Honduras | 10 (4-17) | 0.98 (0.39-1.60) | 2 (1-5) | 0.08 (0.02-0.17) | -8.60 (-8.98 to -8.21) |
| Hungary | 0 (0-0) | 0.01 (0-0.01) | 0 (0-0) | 0 (0-0) | -7.89 (-9.01 to -6.77) |
| Iceland | 0 (0-0) | 0 (0-0) | 0 (0-0) | 0 (0-0) | -0.99 (-1.15 to -0.83) |
| India | 682 (361-956) | 0.34 (0.18-0.47) | 549 (252-795) | 0.15 (0.07-0.21) | -3.37 (-4.6 to -2.12) |
| Indonesia | 196 (83-300) | 0.41 (0.17-0.63) | 30 (12-52) | 0.04 (0.02-0.07) | -7.4 (-7.57 to -7.24) |
| Iran (Islamic Republic of) | 16 (7-25) | 0.12 (0.05-0.20) | 1 (1-2) | 0.01 (0-0.01) | -9.02 (-9.64 to -8.4) |
| Iraq | 1 (0-2) | 0.03 (0.01-0.05) | 1 (0-1) | 0 (0-0.01) | -5.48 (-6.05 to -4.91) |
| Ireland | 0 (0-0) | 0 (0-0) | 0 (0-0) | 0 (0-0) | -4.77 (-5.36 to -4.17) |
| Israel | 0 (0-0) | 0.02 (0.01-0.04) | 0 (0-0) | 0 (0-0) | -6.06 (-6.44 to -5.67) |
| Italy | 0 (0-0) | 0 (0-0) | 0 (0-0) | 0 (0-0) | -0.67 (-1.09 to -0.25) |
| Jamaica | 0 (0-0) | 0.05 (0.02-0.07) | 0 (0-0) | 0.02 (0.01-0.04) | -4.27 (-5.01 to -3.53) |
| Japan | 0 (0-0) | 0 (0-0) | 0 (0-0) | 0 (0-0) | -1.96 (-2.15 to -1.78) |
| Jordan | 3 (1-5) | 0.35 (0.14-0.58) | 0 (0-1) | 0.01 (0-0.02) | -11.4 (-11.85 to -10.95) |
| Kazakhstan | 0 (0-0) | 0 (0-0) | 0 (0-0) | 0 (0-0) | 3.55 (2.16 to 4.95) |
| Kenya | 66 (26-105) | 1.27 (0.51-2.04) | 35 (15-60) | 0.27 (0.11-0.45) | -4.44 (-5.02 to -3.86) |
| Kiribati | 0 (0-0) | 0.17 (0.07-0.28) | 0 (0-0) | 0.21 (0.08-0.39) | 1.41 (1.07 to 1.75) |
| Kuwait | 0 (0-0) | 0 (0-0) | 0 (0-0) | 0 (0-0) | -5.62 (-6.7 to -4.53) |
| Kyrgyzstan | 1 (0-1) | 0.08 (0.04-0.11) | 1 (0-1) | 0.04 (0.02-0.06) | -0.87 (-1.49 to -0.24) |
| Lao People's Democratic Republic | 14 (6-22) | 1.42 (0.62-2.26) | 2 (1-4) | 0.11 (0.04-0.20) | -8.75 (-9.16 to -8.34) |
| Latvia | 0 (0-0) | 0.02 (0.01-0.04) | 0 (0-0) | 0 (0-0) | -8.18 (-8.61 to -7.75) |
| Lebanon | 1 (0-1) | 0.07 (0.02-0.13) | 0 (0-0) | 0.01 (0-0.01) | -8.03 (-8.16 to -7.9) |
| Lesotho | 6 (3-11) | 1.70 (0.69-2.85) | 1 (1-3) | 0.29 (0.11-0.52) | -4.94 (-5.59 to -4.29) |
| Liberia | 12 (6-18) | 2.19 (1.07-3.29) | 6 (3-10) | 0.42 (0.20-0.73) | -6.86 (-7.81 to -5.91) |
| Libya | 1 (0-1) | 0.07 (0.03-0.13) | 0 (0-0) | 0.01 (0-0.02) | -5.49 (-6.33 to -4.65) |
| Lithuania | 0 (0-0) | 0.03 (0.01-0.05) | 0 (0-0) | 0 (0-0) | -7.37 (-7.79 to -6.96) |
| Luxembourg | 0 (0-0) | 0.01 (0-0.01) | 0 (0-0) | 0 (0-0) | -6.85 (-7.3 to -6.39) |
| Madagascar | 41 (18-65) | 1.54 (0.65-2.41) | 61 (25-100) | 0.84 (0.35-1.38) | -2.14 (-2.72 to -1.56) |
| Malawi | 34 (19-48) | 1.52 (0.84-2.14) | 26 (11-44) | 0.51 (0.22-0.87) | -3.75 (-4.5 to -3.00) |
| Malaysia | 6 (2-11) | 0.14 (0.06-0.24) | 2 (1-3) | 0.02 (0.01-0.03) | -6.49 (-6.91 to -6.06) |
| Maldives | 0 (0-0) | 0.24 (0.11-0.37) | 0 (0-0) | 0.01 (0-0.01) | -11.34 (-12 to -10.67) |
| Mali | 78 (41-109) | 4.06 (2.13-5.69) | 70 (33-111) | 1.29 (0.61-2.03) | -3.92 (-4.19 to -3.65) |
| Malta | 0 (0-0) | 0 (0-0) | 0 (0-0) | 0 (0-0) | -2.71 (-3.33 to -2.08) |
| Marshall Islands | 0 (0-0) | 0.26 (0.10-0.58) | 0 (0-0) | 0.64 (0.24-1.36) | 2.74 (1.86 to 3.62) |
| Mauritania | 12 (6-17) | 2.48 (1.19-3.60) | 3 (1-5) | 0.24 (0.10-0.45) | -6.62 (-6.82 to -6.42) |
| Mauritius | 0 (0-0) | 0.03 (0.01-0.04) | 0 (0-0) | 0.01 (0-0.02) | -0.93 (-1.87 to 0.02) |
| Mexico | 11 (4-18) | 0.05 (0.02-0.08) | 2 (1-4) | 0.01 (0-0.01) | -6.59 (-6.73 to -6.46) |
| Micronesia (Federated States of) | 0 (0-0) | 0.10 (0.04-0.17) | 0 (0-0) | 0.03 (0.01-0.06) | -3.06 (-3.56 to -2.56) |
| Monaco | 0 (0-0) | 0 (0-0) | 0 (0-0) | 0 (0-0) | -2.22 (-2.29 to -2.14) |
| Mongolia | 2 (1-4) | 0.44 (0.19-0.69) | 1 (0-1) | 0.06 (0.02-0.10) | -6.84 (-7.31 to -6.38) |
| Montenegro | 0 (0-0) | 0 (0-0) | 0 (0-0) | 0 (0-0) | -2.20 (-2.84 to -1.55) |
| Morocco | 47 (20-74) | 0.75 (0.32-1.17) | 4 (1-9) | 0.04 (0.01-0.09) | -8.98 (-9.35 to -8.61) |
| Mozambique | 35 (15-56) | 1.12 (0.48-1.76) | 18 (8-31) | 0.24 (0.11-0.40) | -4.42 (-4.71 to -4.13) |
| Myanmar | 35 (15-56) | 0.34 (0.15-0.54) | 13 (7-22) | 0.09 (0.04-0.15) | -4.25 (-4.6 to -3.90) |
| Namibia | 4 (2-6) | 1.12 (0.51-1.75) | 1 (0-2) | 0.18 (0.06-0.36) | -5.98 (-6.63 to -5.33) |
| Nauru | 0 (0-0) | 0.08 (0.03-0.17) | 0 (0-0) | 0.06 (0.02-0.13) | -0.39 (-1.13 to 0.34) |
| Nepal | 32 (15-49) | 0.71 (0.33-1.08) | 5 (2-9) | 0.05 (0.02-0.10) | -8.70 (-8.98 to -8.42) |
| Netherlands | 0 (0-0) | 0 (0-0) | 0 (0-0) | 0 (0-0) | -6.21 (-6.5 to -5.92) |
| New Zealand | 0 (0-0) | 0 (0-0) | 0 (0-0) | 0 (0-0) | -4.24 (-5.02 to -3.45) |
| Nicaragua | 2 (1-4) | 0.24 (0.11-0.40) | 0 (0-1) | 0.03 (0.01-0.04) | -7.54 (-7.76 to -7.32) |
| Niger | 55 (25-82) | 3.15 (1.45-4.74) | 100 (39-174) | 1.87 (0.73-3.27) | -1.88 (-2.07 to -1.70) |
| Nigeria | 514 (228-837) | 2.54 (1.13-4.13) | 606 (256-1014) | 1.05 (0.45-1.77) | -3.09 (-3.45 to -2.72) |
| Niue | 0 (0-0) | 0.03 (0.01-0.06) | 0 (0-0) | 0.08 (0.03-0.16) | 4.74 (3.89 to 5.60) |
| North Macedonia | 0 (0-0) | 0.02 (0.01-0.04) | 0 (0-0) | 0 (0-0) | -7.70 (-8.19 to -7.21) |
| Northern Mariana Islands | 0 (0-0) | 0.02 (0.01-0.03) | 0 (0-0) | 0.01 (0-0.02) | -0.99 (-1.53 to -0.44) |
| Norway | 0 (0-0) | 0 (0-0) | 0 (0-0) | 0 (0-0) | -4.56 (-8.21 to -0.77) |
| Oman | 0 (0-0) | 0.05 (0.02-0.11) | 0 (0-0) | 0 (0-0.01) | -8.24 (-8.87 to -7.60) |
| Pakistan | 232 (101-365) | 0.98 (0.43-1.54) | 103 (41-192) | 0.17 (0.07-0.32) | -6.18 (-6.58 to -5.78) |
| Palau | 0 (0-0) | 0.06 (0.02-0.11) | 0 (0-0) | 0.04 (0.02-0.07) | -0.21 (-0.71 to 0.30) |
| Palestine | 0 (0-0) | 0.04 (0.02-0.08) | 0 (0-0) | 0 (0-0.01) | -6.88 (-7.52 to -6.24) |
| Panama | 1 (0-1) | 0.13 (0.06-0.20) | 0 (0-1) | 0.03 (0.01-0.05) | -3.95 (-4.52 to -3.38) |
| Papua New Guinea | 5 (2-9) | 0.54 (0.20-0.94) | 13 (6-21) | 0.49 (0.22-0.8) | -0.08 (-0.23 to 0.08) |
| Paraguay | 4 (2-6) | 0.39 (0.18-0.59) | 2 (1-3) | 0.09 (0.04-0.15) | -4.22 (-4.50 to -3.93) |
| Peru | 35 (16-54) | 0.64 (0.30-0.99) | 10 (4-17) | 0.10 (0.04-0.18) | -7.22 (-8.04 to -6.39) |
| Philippines | 17 (7-26) | 0.11 (0.05-0.17) | 7 (3-12) | 0.02 (0.01-0.04) | -4.58 (-5.04 to -4.12) |
| Poland | 1 (0-1) | 0.01 (0-0.01) | 0 (0-0) | 0 (0-0) | -11.78 (-12.89 to -10.64) |
| Portugal | 0 (0-0) | 0 (0-0) | 0 (0-0) | 0 (0-0) | -5.08 (-5.61 to -4.55) |
| Puerto Rico | 0 (0-0) | 0.03 (0.01-0.04) | 0 (0-0) | 0.01 (0-0.01) | -4.9 (-5.86 to -3.93) |
| Qatar | 0 (0-0) | 0.02 (0.01-0.04) | 0 (0-0) | 0 (0-0) | -8.52 (-8.89 to -8.16) |
| Republic of Côte d'Ivoire | 30 (14-50) | 1.10 (0.50-1.83) | 21 (8-39) | 0.32 (0.12-0.58) | -3.35 (-3.74 to -2.95) |
| Republic of Korea | 3 (1-6) | 0.03 (0.01-0.04) | 0 (0-0) | 0 (0-0) | -9.63 (-10.67 to -8.57) |
| Republic of Moldova | 1 (0-1) | 0.08 (0.03-0.12) | 0 (0-0) | 0 (0-0.01) | -8.37 (-8.82 to -7.92) |
| Romania | 4 (2-7) | 0.08 (0.03-0.12) | 0 (0-0) | 0 (0-0.01) | -9.04 (-9.42 to -8.67) |
| Russian Federation | 9 (4-15) | 0.03 (0.01-0.04) | 1 (0-1) | 0 (0-0) | -7.06 (-7.41 to -6.72) |
| Rwanda | 82 (32-140) | 5.05 (1.98-8.61) | 30 (11-53) | 0.85 (0.32-1.50) | -6.85 (-7.65 to -6.04) |
| Saint Kitts and Nevis | 0 (0-0) | 0.43 (0.20-0.62) | 0 (0-0) | 0.08 (0.04-0.13) | -4.2 (-5.35 to -3.03) |
| Saint Lucia | 0 (0-0) | 0.04 (0.02-0.06) | 0 (0-0) | 0.01 (0-0.01) | -4.01 (-5.04 to -2.96) |
| Saint Vincent and the Grenadines | 0 (0-0) | 0 (0-0.01) | 0 (0-0) | 0 (0-0) | -2.95 (-3.53 to -2.38) |
| Samoa | 0 (0-0) | 0.02 (0.01-0.04) | 0 (0-0) | 0.01 (0-0.02) | -0.73 (-1.6 to 0.15) |
| San Marino | 0 (0-0) | 0 (0-0) | 0 (0-0) | 0 (0-0) | -2.7 (-2.87 to -2.54) |
| Sao Tome and Principe | 0 (0-0) | 0.69 (0.29-1.16) | 0 (0-0) | 0.04 (0.02-0.09) | -9.75 (-10.53 to -8.97) |
| Saudi Arabia | 5 (2-8) | 0.14 (0.05-0.26) | 1 (0-1) | 0.01 (0-0.01) | -7.50 (-8.11 to -6.89) |
| Senegal | 24 (12-35) | 1.40 (0.69-2.04) | 15 (7-24) | 0.38 (0.18-0.62) | -3.45 (-3.88 to -3.03) |
| Serbia | 0 (0-1) | 0.01 (0-0.02) | 0 (0-0) | 0 (0-0) | -7.14 (-7.51 to -6.78) |
| Seychelles | 0 (0-0) | 0.02 (0.01-0.03) | 0 (0-0) | 0.01 (0-0.01) | -2.70 (-3.12 to -2.27) |
| Sierra Leone | 11 (5-18) | 1.07 (0.46-1.80) | 12 (5-22) | 0.55 (0.2-0.98) | -2.27 (-2.92 to -1.62) |
| Singapore | 0 (0-0) | 0 (0-0) | 0 (0-0) | 0 (0-0) | -7.64 (-8.43 to -6.83) |
| Slovakia | 0 (0-0) | 0 (0-0) | 0 (0-0) | 0 (0-0) | -6.08 (-6.90 to -5.25) |
| Slovenia | 0 (0-0) | 0 (0-0) | 0 (0-0) | 0 (0-0) | -2.04 (-2.42 to -1.65) |
| Solomon Islands | 0 (0-1) | 0.52 (0.18-0.96) | 1 (0-1) | 0.48 (0.21-0.83) | -0.22 (-0.35 to -0.08) |
| Somalia | 92 (43-145) | 5.42 (2.53-8.58) | 144 (63-248) | 2.98 (1.30-5.12) | -2.11 (-2.26 to -1.95) |
| South Africa | 57 (23-94) | 0.59 (0.23-0.98) | 9 (4-14) | 0.06 (0.02-0.09) | -6.70 (-8.2 to -5.17) |
| South Sudan | 26 (10-46) | 2 (0.75-3.53) | 26 (11-49) | 1.14 (0.46-2.1) | -2.11 (-2.27 to -1.96) |
| Spain | 0 (0-0) | 0 (0-0) | 0 (0-0) | 0 (0-0) | -5.14 (-5.58 to -4.70) |
| Sri Lanka | 2 (1-3) | 0.04 (0.02-0.07) | 0 (0-0) | 0 (0-0.01) | -7.93 (-8.12 to -7.73) |
| Sudan | 129 (56-207) | 2.75 (1.19-4.42) | 42 (15-84) | 0.37 (0.13-0.74) | -6.24 (-6.78 to -5.69) |
| Suriname | 0 (0-0) | 0.08 (0.03-0.14) | 0 (0-0) | 0.04 (0.02-0.07) | -2.12 (-2.77 to -1.46) |
| Sweden | 0 (0-0) | 0 (0-0) | 0 (0-0) | 0 (0-0) | -4.14 (-4.79 to -3.49) |
| Switzerland | 0 (0-0) | 0 (0-0) | 0 (0-0) | 0 (0-0) | -4.29 (-4.63 to -3.95) |
| Syrian Arab Republic | 5 (2-8) | 0.17 (0.07-0.27) | 1 (0-1) | 0.02 (0.01-0.04) | -7.67 (-8.19 to -7.16) |
| Taiwan (Province of China) | 0 (0-0) | 0 (0-0) | 0 (0-0) | 0 (0-0) | -5.12 (-5.56 to -4.69) |
| Tajikistan | 2 (1-2) | 0.13 (0.05-0.20) | 1 (0-2) | 0.04 (0.02-0.07) | -3.98 (-4.49 to -3.46) |
| Thailand | 2 (1-4) | 0.02 (0.01-0.03) | 2 (1-4) | 0.01 (0.01-0.03) | 1.54 (0.33 to 2.76) |
| Timor-Leste | 4 (2-7) | 2.33 (0.88-3.83) | 1 (0-2) | 0.38 (0.14-0.72) | -6.76 (-7.27 to -6.26) |
| Togo | 13 (6-21) | 1.54 (0.69-2.49) | 8 (3-13) | 0.36 (0.14-0.6) | -4.67 (-5.19 to -4.15) |
| Tokelau | 0 (0-0) | 0.08 (0.03-0.15) | 0 (0-0) | 0.04 (0.02-0.08) | -2.75 (-3.3 to -2.21) |
| Tonga | 0 (0-0) | 0.04 (0.01-0.07) | 0 (0-0) | 0.01 (0-0.02) | -2.57 (-3.37 to -1.77) |
| Trinidad and Tobago | 0 (0-0) | 0.06 (0.03-0.08) | 0 (0-0) | 0.03 (0.01-0.05) | -1.34 (-1.89 to -0.80) |
| Tunisia | 2 (1-3) | 0.09 (0.03-0.15) | 0 (0-1) | 0.01 (0-0.02) | -5.89 (-6.36 to -5.41) |
| Turkey | 26 (10-44) | 0.18 (0.07-0.31) | 2 (1-4) | 0.01 (0-0.02) | -9.25 (-10.06 to -8.44) |
| Turkmenistan | 0 (0-0) | 0.03 (0.02-0.05) | 0 (0-0) | 0.02 (0.01-0.04) | -1.72 (-2.18 to -1.26) |
| Tuvalu | 0 (0-0) | 0.17 (0.06-0.39) | 0 (0-0) | 0.07 (0.02-0.14) | -2.4 (-3.08 to -1.72) |
| Uganda | 57 (26-92) | 1.49 (0.67-2.40) | 42 (16-76) | 0.41 (0.16-0.73) | -4.51 (-5.05 to -3.97) |
| Ukraine | 1 (1-2) | 0.01 (0-0.02) | 0 (0-0) | 0 (0-0) | -5.47 (-5.93 to -5.00) |
| United Arab Emirates | 0 (0-0) | 0.01 (0.01-0.03) | 0 (0-0) | 0 (0-0.01) | -4.43 (-5.17 to -3.68) |
| United Kingdom | 0 (0-0) | 0 (0-0) | 0 (0-0) | 0 (0-0) | 1.77 (0.67 to 2.87) |
| United Republic of Tanzania | 78 (42-107) | 1.30 (0.71-1.78) | 61 (27-98) | 0.41 (0.18-0.66) | -3.44 (-4.03 to -2.85) |
| United States of America | 2 (1-3) | 0 (0-0) | 1 (1-2) | 0 (0-0) | -0.49 (-0.96 to -0.03) |
| United States Virgin Islands | 0 (0-0) | 0.01 (0.01-0.03) | 0 (0-0) | 0 (0-0.01) | -3.72 (-4.01 to -3.44) |
| Uruguay | 1 (0-1) | 0.10 (0.04-0.15) | 0 (0-0) | 0.02 (0.01-0.04) | -4.34 (-4.72 to -3.96) |
| Uzbekistan | 3 (2-4) | 0.06 (0.03-0.09) | 1 (1-2) | 0.01 (0.01-0.02) | -3.94 (-4.71 to -3.17) |
| Vanuatu | 0 (0-0) | 0.15 (0.05-0.29) | 0 (0-0) | 0.10 (0.04-0.19) | -1.33 (-1.51 to -1.15) |
| Venezuela (Bolivarian Republic of) | 10 (5-16) | 0.22 (0.09-0.32) | 5 (2-9) | 0.08 (0.03-0.13) | -3.15 (-3.87 to -2.42) |
| Viet Nam | 3 (1-6) | 0.02 (0.01-0.04) | 0 (0-1) | 0 (0-0) | -7.63 (-8.34 to -6.91) |
| Yemen | 54 (27-87) | 1.99 (0.97-3.18) | 39 (18-69) | 0.47 (0.21-0.83) | -5.27 (-5.55 to -4.99) |
| Zambia | 19 (8-30) | 1.03 (0.45-1.65) | 15 (6-27) | 0.3 (0.12-0.55) | -4.35 (-4.81 to -3.88) |
| Zimbabwe | 25 (11-40) | 1.06 (0.47-1.68) | 33 (13-57) | 0.81 (0.32-1.39) | 1.37 (0.12 to 2.63) |
| DALYs (Disability-Adjusted Life Years) |  |  |  |  |  |
| Afghanistan | 1716 (663-2909) | 78 (30.11-132.2) | 3113 (1121-5914) | 43.27 (15.58-82.19) | -2.48 (-3.24 to -1.71) |
| Albania | 27 (11-44) | 3.24 (1.34-5.3) | 4 (1-8) | 0.7 (0.23-1.36) | -4.83 (-5.4 to -4.26) |
| Algeria | 2112 (882-3306) | 36.34 (15.18-56.89) | 447 (175-793) | 3.98 (1.56-7.06) | -6.87 (-7.04 to -6.70) |
| American Samoa | 1 (0-1) | 5.53 (2.12-9.21) | 1 (0-1) | 5.41 (2.08-9.52) | 0.23 (0 to 0.47) |
| Andorra | 0 (0-0) | 0.48 (0.16-1.03) | 0 (0-0) | 0.23 (0.06-0.51) | -2.70 (-2.83 to -2.58) |
| Angola | 3234 (1338-5433) | 140.50 (58.12-236) | 1914 (813-3338) | 24.94 (10.59-43.49) | -5.81 (-6.07 to -5.55) |
| Antigua and Barbuda | 1 (1-2) | 7.33 (3.24-10.71) | 1 (0-1) | 2.43 (1.05-3.72) | -2.58 (-3.19 to -1.96) |
| Argentina | 1084 (477-1639) | 13.50 (5.94-20.42) | 351 (139-563) | 2.95 (1.17-4.73) | -3.79 (-4.16 to -3.41) |
| Armenia | 23 (9-38) | 2.67 (1.08-4.38) | 4 (2-7) | 0.59 (0.22-1.01) | -3.89 (-4.44 to -3.33) |
| Australia | 17 (4-40) | 0.38 (0.10-0.90) | 23 (6-51) | 0.38 (0.11-0.85) | 0.83 (0.33 to 1.34) |
| Austria | 17 (5-34) | 0.85 (0.27-1.73) | 12 (3-25) | 0.59 (0.17-1.27) | -1.16 (-1.24 to -1.09) |
| Azerbaijan | 24 (9-47) | 1.26 (0.48-2.53) | 13 (5-27) | 0.48 (0.17-0.99) | -2.48 (-2.81 to -2.15) |
| Bahamas | 2 (1-4) | 3.29 (1.46-5.39) | 1 (1-2) | 1.39 (0.6-2.26) | -2.17 (-2.71 to -1.63) |
| Bahrain | 4 (2-7) | 3.52 (1.49-5.74) | 3 (1-5) | 0.89 (0.34-1.61) | -3.53 (-3.8 to -3.26) |
| Bangladesh | 3932 (1844-5548) | 15.97 (7.49-22.54) | 472 (183-904) | 1.03 (0.4-1.97) | -8.28 (-9.01 to -7.53) |
| Barbados | 5 (2-7) | 6.99 (3.04-10.33) | 2 (1-3) | 2.68 (1.17-4.23) | -2.44 (-3.06 to -1.81) |
| Belarus | 55 (20-93) | 2.16 (0.80-3.66) | 18 (5-36) | 0.83 (0.26-1.68) | -1.79 (-2.27 to -1.32) |
| Belgium | 10 (3-21) | 0.43 (0.14-0.88) | 5 (1-12) | 0.21 (0.06-0.46) | -2.33 (-3.24 to -1.40) |
| Belize | 5 (2-7) | 11.09 (4.90-16.38) | 6 (3-9) | 4.81 (2.11-7.43) | -1.31 (-1.78 to -0.84) |
| Benin | 1293 (574-1991) | 118.06 (52.39-181.79) | 721 (325-1200) | 22.22 (10.02-37) | -5.04 (-5.43 to -4.65) |
| Bermuda | 0 (0-0) | 1.41 (0.55-2.44) | 0 (0-0) | 0.48 (0.15-1.01) | -2.89 (-3.22 to -2.56) |
| Bhutan | 36 (16-60) | 25.14 (11-42.09) | 4 (2-7) | 1.81 (0.8-3.19) | -8.43 (-8.60 to -8.26) |
| Bolivia (Plurinational State of) | 1072 (452-1675) | 70.08 (29.56-109.48) | 352 (143-637) | 11.29 (4.59-20.43) | -6.13 (-6.33 to -5.94) |
| Bosnia and Herzegovina | 34 (14-56) | 2.93 (1.19-4.77) | 3 (1-7) | 0.46 (0.16-0.93) | -6.99 (-7.94 to -6.03) |
| Botswana | 273 (116-483) | 84.86 (35.95-150.01) | 50 (20-81) | 7.39 (2.97-11.86) | -7.05 (-7.61 to -6.49) |
| Brazil | 4407 (2064-6214) | 11.31 (5.3-15.95) | 1779 (780-2687) | 3.03 (1.33-4.58) | -3.38 (-3.72 to -3.04) |
| Brunei Darussalam | 1 (0-1) | 0.98 (0.35-1.79) | 0 (0-1) | 0.28 (0.09-0.56) | -3.97 (-4.25 to -3.69) |
| Bulgaria | 43 (17-74) | 2.10 (0.84-3.57) | 7 (3-14) | 0.51 (0.18-1) | -3.87 (-4.25 to -3.49) |
| Burkina Faso | 2121 (842-3347) | 101.33 (40.23-159.89) | 2316 (1044-3725) | 42.17 (19.01-67.82) | -3.48 (-3.80 to -3.15) |
| Burundi | 2479 (991-4313) | 196.86 (78.67-342.44) | 2082 (800-3642) | 66.58 (25.58-116.45) | -4.24 (-4.65 to -3.83) |
| Cabo Verde | 19 (8-32) | 23.74 (9.68-40.21) | 2 (1-3) | 1.2 (0.47-2.07) | -9.86 (-10.14 to -9.59) |
| Cambodia | 1083 (503-1686) | 43.25 (20.08-67.33) | 250 (101-447) | 5.53 (2.23-9.89) | -7.98 (-8.64 to -7.31) |
| Cameroon | 1402 (573-2240) | 58.92 (24.1-94.16) | 995 (425-1692) | 12.71 (5.43-21.61) | -4.44 (-5.03 to -3.85) |
| Canada | 44 (17-81) | 0.60 (0.23-1.10) | 35 (14-65) | 0.43 (0.16-0.79) | -1.02 (-1.30 to -0.73) |
| Central African Republic | 1120 (464-1834) | 174.75 (72.35-286.06) | 1633 (605-2960) | 118.04 (43.72-213.97) | -1.20 (-1.45 to -0.95) |
| Chad | 3303 (1496-4917) | 248.39 (112.53-369.76) | 6336 (2996-10283) | 163.82 (77.46-265.84) | -1.53 (-1.89 to -1.17) |
| Chile | 337 (144-520) | 9.31 (3.98-14.39) | 69 (22-135) | 1.46 (0.47-2.86) | -5.45 (-6.12 to -4.78) |
| China | 10490 (4125-16821) | 3.26 (1.28-5.22) | 1142 (367-2292) | 0.36 (0.12-0.72) | -6.66 (-7.26 to -6.05) |
| Colombia | 633 (258-1043) | 7.29 (2.97-12.01) | 186 (66-335) | 1.42 (0.5-2.56) | -5.29 (-5.73 to -4.86) |
| Comoros | 55 (20-92) | 52.07 (18.85-87.76) | 25 (10-46) | 12.66 (5.11-23.52) | -5.45 (-5.95 to -4.94) |
| Congo | 446 (190-726) | 79.14 (33.78-128.85) | 308 (134-537) | 21.5 (9.38-37.49) | -4.06 (-4.63 to -3.49) |
| Cook Islands | 0 (0-0) | 1.35 (0.43-2.74) | 0 (0-0) | 0.55 (0.18-1.16) | -3.07 (-3.28 to -2.85) |
| Costa Rica | 67 (28-102) | 8.55 (3.55-13.06) | 21 (8-36) | 1.6 (0.64-2.75) | -5.61 (-5.79 to -5.42) |
| Croatia | 11 (4-20) | 0.91 (0.33-1.7) | 4 (1-8) | 0.45 (0.14-0.92) | -1.84 (-2.08 to -1.59) |
| Cuba | 156 (66-242) | 5.09 (2.16-7.89) | 31 (12-53) | 1.25 (0.5-2.14) | -4.22 (-4.84 to -3.59) |
| Cyprus | 1 (0-3) | 0.72 (0.21-1.71) | 1 (0-2) | 0.21 (0.05-0.48) | -4.08 (-4.55 to -3.60) |
| Czechia | 17 (5-34) | 0.64 (0.21-1.34) | 6 (2-13) | 0.25 (0.07-0.57) | -1.68 (-2.2 to -1.17) |
| Democratic People's Republic of Korea | 107 (40-192) | 1.88 (0.71-3.39) | 52 (20-96) | 0.79 (0.31-1.46) | -3.23 (-3.48 to -2.98) |
| Democratic Republic of the Congo | 10374 (4553-16403) | 121.56 (53.35-192.19) | 21880 (8945-38154) | 102.69 (41.98-179.06) | 0.27 (-0.6 to 1.16) |
| Denmark | 11 (4-21) | 0.85 (0.27-1.63) | 7 (2-16) | 0.57 (0.16-1.24) | -1.78 (-1.95 to -1.61) |
| Djibouti | 99 (44-164) | 100.86 (45.06-167.06) | 99 (35-187) | 30.85 (10.82-58.21) | -3.82 (-4.15 to -3.49) |
| Dominica | 2 (1-3) | 11.53 (5.1-18.09) | 1 (0-2) | 5.51 (2.13-9.32) | -1.8 (-2.52 to -1.08) |
| Dominican Republic | 206 (94-311) | 10.93 (5-16.46) | 96 (42-157) | 3.32 (1.44-5.41) | -2.98 (-3.47 to -2.49) |
| Ecuador | 542 (223-866) | 21.48 (8.86-34.33) | 166 (61-288) | 3.52 (1.29-6.11) | -4.78 (-5.41 to -4.15) |
| Egypt | 3795 (1622-6027) | 28.94 (12.37-45.96) | 647 (246-1079) | 2.49 (0.95-4.16) | -6.81 (-7.42 to -6.2) |
| El Salvador | 373 (136-629) | 28.46 (10.34-48) | 24 (8-45) | 1.37 (0.48-2.53) | -8.77 (-10.28 to -7.24) |
| Equatorial Guinea | 245 (104-414) | 248.21 (105.39-418.74) | 21 (8-42) | 5.73 (2.18-11.42) | -12.92 (-13.34 to -12.49) |
| Eritrea | 2531 (1194-3716) | 321 (151.38-471.26) | 1383 (563-2465) | 83.54 (34.02-148.91) | -3.89 (-4.20 to -3.58) |
| Estonia | 8 (3-13) | 2.01 (0.75-3.46) | 2 (1-5) | 0.81 (0.25-1.72) | -2.07 (-2.53 to -1.61) |
| Eswatini | 57 (22-95) | 29.17 (11.23-48.86) | 27 (9-54) | 8.54 (2.85-17.09) | -3.01 (-3.53 to -2.49) |
| Ethiopia | 20343 (8317-30577) | 180.28 (73.71-270.98) | 4903 (1941-8301) | 17.69 (7-29.95) | -8.37 (-9.00 to -7.73) |
| Fiji | 11 (4-20) | 5.84 (2.26-10.18) | 7 (3-13) | 3.23 (1.28-5.7) | -1.81 (-2.01 to -1.60) |
| Finland | 9 (3-16) | 0.71 (0.25-1.23) | 3 (1-7) | 0.29 (0.10-0.63) | -1.99 (-2.37 to -1.60) |
| France | 68 (20-142) | 0.47 (0.14-0.98) | 44 (12-102) | 0.31 (0.08-0.72) | -0.98 (-1.12 to -0.84) |
| Gabon | 100 (48-151) | 45.25 (21.61-68.04) | 21 (9-38) | 4.29 (1.78-7.78) | -7.59 (-7.96 to -7.22) |
| Gambia | 290 (135-467) | 127.91 (59.69-205.94) | 250 (109-417) | 40.68 (17.71-67.73) | -3.66 (-3.94 to -3.38) |
| Georgia | 38 (16-61) | 2.79 (1.17-4.42) | 9 (4-15) | 1.15 (0.46-1.88) | -1.84 (-2.38 to -1.29) |
| Germany | 404 (180-656) | 2.08 (0.93-3.38) | 135 (44-265) | 0.79 (0.26-1.56) | -2.33 (-2.79 to -1.86) |
| Ghana | 2493 (1036-4129) | 70.68 (29.38-117.08) | 1037 (480-1716) | 11.35 (5.26-18.79) | -5.68 (-5.85 to -5.52) |
| Greece | 15 (6-29) | 0.58 (0.22-1.14) | 5 (2-10) | 0.23 (0.07-0.46) | -2.68 (-3.02 to -2.33) |
| Greenland | 0 (0-0) | 1.15 (0.35-2.52) | 0 (0-0) | 0.6 (0.2-1.26) | -1.87 (-2.08 to -1.66) |
| Grenada | 0 (0-1) | 2.27 (0.86-4.22) | 0 (0-0) | 0.92 (0.32-1.8) | -2.78 (-2.87 to -2.68) |
| Guam | 1 (0-1) | 2.03 (0.75-3.71) | 0 (0-1) | 1.09 (0.38-2.1) | -1.86 (-2.24 to -1.47) |
| Guatemala | 1458 (662-2108) | 79.35 (36.05-114.77) | 517 (227-796) | 11.76 (5.16-18.11) | -6.22 (-6.94 to -5.49) |
| Guinea | 2685 (1248-4021) | 196.87 (91.47-294.76) | 1973 (800-3457) | 59.46 (24.12-104.17) | -3.53 (-3.68 to -3.39) |
| Guinea-Bissau | 205 (89-347) | 88.04 (38.26-148.89) | 145 (67-257) | 27.56 (12.75-48.85) | -3.34 (-3.56 to -3.13) |
| Guyana | 18 (9-29) | 9 (4.26-14.25) | 8 (3-13) | 3.89 (1.56-6.41) | -2.36 (-2.63 to -2.08) |
| Haiti | 1905 (878-2924) | 123.81 (57.05-190.04) | 2628 (1236-4347) | 74.23 (34.91-122.8) | -1.37 (-1.68 to -1.06) |
| Honduras | 675 (274-1098) | 64.01 (25.95-104.04) | 168 (55-329) | 5.92 (1.92-11.6) | -8.23 (-8.58 to -7.87) |
| Hungary | 27 (10-47) | 1.05 (0.41-1.87) | 8 (2-16) | 0.36 (0.11-0.76) | -2.95 (-3.48 to -2.41) |
| Iceland | 0 (0-1) | 0.6 (0.15-1.35) | 0 (0-1) | 0.31 (0.08-0.72) | -1.95 (-2.07 to -1.83) |
| India | 48765 (25588-68498) | 24.15 (12.67-33.93) | 35884 (16660-51730) | 9.49 (4.4-13.67) | -3.65 (-4.82 to -2.47) |
| Indonesia | 12505 (5350-19000) | 26.09 (11.16-39.65) | 2304 (897-3893) | 3.06 (1.19-5.17) | -6.87 (-7.00 to -6.73) |
| Iran (Islamic Republic of) | 1185 (496-1887) | 9.37 (3.92-14.92) | 196 (70-357) | 0.84 (0.3-1.54) | -6.56 (-7.17 to -5.95) |
| Iraq | 162 (60-292) | 3.98 (1.47-7.2) | 132 (47-255) | 1.25 (0.45-2.42) | -3.77 (-4.07 to -3.48) |
| Ireland | 7 (2-13) | 0.74 (0.25-1.51) | 4 (1-10) | 0.38 (0.11-0.84) | -1.85 (-2.05 to -1.65) |
| Israel | 29 (11-49) | 2.37 (0.89-3.98) | 21 (7-43) | 0.94 (0.29-1.93) | -2.41 (-2.62 to -2.21) |
| Italy | 51 (14-115) | 0.35 (0.10-0.80) | 27 (8-54) | 0.22 (0.07-0.45) | -1.46 (-1.65 to -1.27) |
| Jamaica | 27 (12-42) | 4.50 (2.07-7.03) | 16 (7-26) | 2.07 (0.88-3.38) | -3.97 (-4.50 to -3.43) |
| Japan | 117 (40-232) | 0.36 (0.12-0.72) | 65 (21-131) | 0.26 (0.08-0.53) | -1.47 (-1.65 to -1.28) |
| Jordan | 191 (78-305) | 22.95 (9.3-36.58) | 46 (19-77) | 1.47 (0.61-2.5) | -9.47 (-9.83 to -9.11) |
| Kazakhstan | 35 (13-75) | 0.86 (0.31-1.83) | 32 (12-62) | 0.68 (0.25-1.3) | 0.76 (0.16 to 1.37) |
| Kenya | 4144 (1658-6628) | 80.01 (32.02-127.96) | 2247 (943-3722) | 16.93 (7.11-28.04) | -4.43 (-4.99 to -3.88) |
| Kiribati | 2 (1-3) | 11.89 (5.21-18.72) | 4 (2-7) | 12.38 (5.01-22.3) | 0.68 (0.38 to 0.97) |
| Kuwait | 5 (2-11) | 1.26 (0.43-2.59) | 5 (2-11) | 0.36 (0.12-0.73) | -4.29 (-4.78 to -3.8) |
| Kyrgyzstan | 60 (28-90) | 5.7 (2.69-8.56) | 51 (23-78) | 2.95 (1.34-4.53) | -0.86 (-1.43 to -0.29) |
| Lao People's Democratic Republic | 878 (382-1400) | 90.69 (39.43-144.68) | 149 (62-277) | 7.52 (3.14-13.95) | -8.36 (-8.72 to -8.00) |
| Latvia | 16 (6-27) | 2.53 (0.98-4.17) | 3 (1-7) | 0.87 (0.25-1.79) | -2.5 (-3.08 to -1.93) |
| Lebanon | 44 (16-80) | 5.86 (2.09-10.66) | 12 (4-22) | 0.81 (0.3-1.47) | -6.23 (-6.33 to -6.14) |
| Lesotho | 395 (157-661) | 104.33 (41.51-174.61) | 94 (36-167) | 18.51 (7.15-32.99) | -4.74 (-5.38 to -4.09) |
| Liberia | 746 (366-1120) | 134.43 (65.99-201.92) | 393 (191-689) | 28.2 (13.68-49.38) | -6.43 (-7.34 to -5.51) |
| Libya | 57 (22-93) | 6.29 (2.42-10.17) | 17 (7-31) | 0.88 (0.34-1.55) | -5.57 (-6.06 to -5.08) |
| Lithuania | 27 (10-46) | 2.98 (1.1-4.95) | 5 (2-11) | 0.93 (0.29-1.89) | -3.15 (-3.66 to -2.65) |
| Luxembourg | 1 (0-2) | 0.86 (0.31-1.55) | 0 (0-1) | 0.31 (0.09-0.61) | -3.27 (-3.39 to -3.15) |
| Madagascar | 2570 (1098-4043) | 95.41 (40.78-150.1) | 3774 (1559-6167) | 52.11 (21.52-85.14) | -2.16 (-2.73 to -1.58) |
| Malawi | 1987 (1106-2812) | 88.01 (48.99-124.53) | 1543 (654-2642) | 30.8 (13.05-52.74) | -3.6 (-4.35 to -2.84) |
| Malaysia | 428 (170-707) | 9.54 (3.79-15.78) | 150 (66-236) | 1.78 (0.78-2.8) | -5.72 (-6.12 to -5.32) |
| Maldives | 8 (4-12) | 16.69 (7.93-24.98) | 1 (1-2) | 1.01 (0.44-1.8) | -8.60 (-9.30 to -7.89) |
| Mali | 4595 (2430-6369) | 239.47 (126.63-331.89) | 4277 (2049-6749) | 78.56 (37.64-123.97) | -3.79 (-4.05 to -3.52) |
| Malta | 1 (0-2) | 0.84 (0.27-1.82) | 0 (0-1) | 0.47 (0.13-1.11) | -1.65 (-1.78 to -1.52) |
| Marshall Islands | 2 (1-3) | 16.25 (6.09-33.71) | 5 (2-10) | 33.71 (12.92-70.7) | 2.34 (1.56 to 3.13) |
| Mauritania | 704 (339-1026) | 149.81 (72.15-218.29) | 163 (68-297) | 15.19 (6.3-27.66) | -6.54 (-6.73 to -6.35) |
| Mauritius | 7 (3-12) | 2.41 (1.05-3.87) | 3 (1-5) | 1.04 (0.42-1.66) | -1.72 (-2.24 to -1.19) |
| Mexico | 1014 (360-1719) | 4.63 (1.64-7.85) | 418 (145-802) | 1.19 (0.41-2.29) | -4.06 (-4.33 to -3.78) |
| Micronesia (Federated States of) | 2 (1-3) | 7.91 (3.16-13.86) | 1 (0-1) | 2.69 (1.07-4.8) | -2.96 (-3.31 to -2.61) |
| Monaco | 0 (0-0) | 0.48 (0.15-1) | 0 (0-0) | 0.35 (0.11-0.76) | -1.26 (-1.39 to -1.12) |
| Mongolia | 146 (64-227) | 28.61 (12.56-44.27) | 35 (13-60) | 4.12 (1.57-7.1) | -6.63 (-7.11 to -6.15) |
| Montenegro | 1 (0-2) | 0.71 (0.22-1.44) | 1 (0-1) | 0.42 (0.13-0.88) | -1.60 (-1.65 to -1.55) |
| Morocco | 2979 (1272-4609) | 47.31 (20.19-73.18) | 309 (113-554) | 3.19 (1.17-5.72) | -8.51 (-8.83 to -8.19) |
| Mozambique | 2190 (942-3423) | 69.41 (29.84-108.48) | 1176 (559-1932) | 15.49 (7.36-25.45) | -4.31 (-4.59 to -4.02) |
| Myanmar | 2318 (1017-3631) | 22.24 (9.76-34.83) | 916 (464-1443) | 6.06 (3.07-9.55) | -4.15 (-4.47 to -3.83) |
| Namibia | 237 (108-367) | 69.82 (31.9-108.18) | 76 (27-152) | 11.53 (4.15-22.92) | -5.78 (-6.39 to -5.16) |
| Nauru | 0 (0-0) | 7.01 (2.84-12.67) | 0 (0-0) | 4.93 (1.92-9.33) | -0.72 (-1.35 to -0.10) |
| Nepal | 2100 (1006-3195) | 45.97 (22.03-69.95) | 381 (158-679) | 4.2 (1.74-7.49) | -8.10 (-8.34 to -7.86) |
| Netherlands | 24 (7-49) | 0.61 (0.19-1.22) | 14 (4-31) | 0.37 (0.11-0.83) | -1.56 (-1.66 to -1.45) |
| New Zealand | 12 (3-24) | 1.31 (0.38-2.69) | 11 (3-23) | 0.94 (0.28-1.95) | -0.97 (-1.12 to -0.83) |
| Nicaragua | 159 (71-258) | 17.67 (7.87-28.59) | 46 (17-80) | 2.51 (0.91-4.4) | -6.70 (-6.92 to -6.49) |
| Niger | 3334 (1532-4943) | 191.64 (88.07-284.18) | 6187 (2448-10768) | 116.05 (45.91-201.99) | -1.82 (-2.00 to -1.64) |
| Nigeria | 32509 (14558-52983) | 160.52 (71.89-261.62) | 38284 (16216-63594) | 66.67 (28.24-110.75) | -3.11 (-3.47 to -2.76) |
| Niue | 0 (0-0) | 2.77 (1.02-5.2) | 0 (0-0) | 4.46 (1.65-8.47) | 2.26 (1.63 to 2.89) |
| North Macedonia | 11 (4-19) | 2.16 (0.77-3.65) | 2 (1-5) | 0.45 (0.16-0.93) | -5.17 (-5.55 to -4.79) |
| Northern Mariana Islands | 0 (0-1) | 2.14 (0.8-3.7) | 0 (0-0) | 1.21 (0.48-2.15) | -1.82 (-2.21 to -1.44) |
| Norway | 7 (2-15) | 0.68 (0.2-1.43) | 5 (1-11) | 0.41 (0.10-0.91) | -2.03 (-2.47 to -1.58) |
| Oman | 18 (7-32) | 5.28 (2.08-9.45) | 8 (3-16) | 0.78 (0.28-1.54) | -5.42 (-5.71 to -5.13) |
| Pakistan | 14830 (6532-23096) | 62.69 (27.61-97.64) | 7104 (2989-12605) | 11.73 (4.93-20.81) | -5.93 (-6.29 to -5.56) |
| Palau | 0 (0-0) | 4.57 (1.71-8.14) | 0 (0-0) | 2.97 (1.26-5.31) | -0.63 (-1.03 to -0.23) |
| Palestine | 21 (9-38) | 4.65 (1.95-8.6) | 14 (5-27) | 1.05 (0.35-2.1) | -5.09 (-5.47 to -4.71) |
| Panama | 61 (27-91) | 10.02 (4.47-14.95) | 33 (14-53) | 3.11 (1.35-4.96) | -3.26 (-3.67 to -2.84) |
| Papua New Guinea | 336 (126-582) | 34.83 (13.06-60.42) | 769 (350-1239) | 29.39 (13.36-47.36) | -0.33 (-0.47 to -0.19) |
| Paraguay | 243 (116-367) | 25.9 (12.35-39) | 130 (52-213) | 6.84 (2.76-11.21) | -3.96 (-4.22 to -3.70) |
| Peru | 2238 (1035-3388) | 41.19 (19.05-62.37) | 715 (289-1211) | 7.43 (3.01-12.59) | -6.65 (-7.41 to -5.88) |
| Philippines | 1260 (543-1928) | 8.12 (3.5-12.42) | 675 (265-1120) | 2.3 (0.91-3.82) | -3.80 (-4.15 to -3.46) |
| Poland | 142 (53-256) | 1.51 (0.57-2.72) | 38 (11-76) | 0.43 (0.13-0.86) | -3.51 (-3.84 to -3.17) |
| Portugal | 18 (6-38) | 0.71 (0.25-1.5) | 7 (2-14) | 0.29 (0.10-0.62) | -2.87 (-3.03 to -2.70) |
| Puerto Rico | 27 (11-45) | 2.85 (1.11-4.73) | 6 (2-9) | 0.74 (0.27-1.26) | -4.19 (-4.48 to -3.90) |
| Qatar | 2 (1-4) | 2.82 (1.09-4.84) | 3 (1-6) | 0.56 (0.19-1.14) | -4.64 (-4.94 to -4.34) |
| Republic of Côte d'Ivoire | 1868 (846-3056) | 68.25 (30.93-111.68) | 1357 (528-2417) | 20.38 (7.93-36.3) | -3.3 (-3.68 to -2.92) |
| Republic of Korea | 247 (103-388) | 1.95 (0.82-3.07) | 15 (5-29) | 0.13 (0.04-0.25) | -8.16 (-9.06 to -7.26) |
| Republic of Moldova | 72 (31-112) | 6.4 (2.72-9.92) | 8 (3-16) | 0.94 (0.35-1.76) | -5.58 (-6.00 to -5.16) |
| Romania | 319 (127-504) | 5.67 (2.26-8.96) | 33 (12-61) | 0.8 (0.29-1.5) | -5.56 (-6.04 to -5.07) |
| Russian Federation | 950 (361-1574) | 2.57 (0.97-4.25) | 328 (108-638) | 0.97 (0.32-1.88) | -2.27 (-2.62 to -1.91) |
| Rwanda | 4725 (1867-8032) | 291.2 (115.08-495.06) | 1719 (643-3020) | 48.82 (18.26-85.75) | -6.78 (-7.58 to -5.98) |
| Saint Kitts and Nevis | 3 (1-4) | 27.53 (12.67-40.27) | 1 (0-1) | 5.69 (2.49-8.73) | -4.08 (-5.09 to -3.05) |
| Saint Lucia | 1 (1-2) | 4.12 (1.85-6.64) | 0 (0-1) | 0.99 (0.41-1.69) | -3.86 (-4.41 to -3.30) |
| Saint Vincent and the Grenadines | 0 (0-1) | 1.83 (0.71-3.56) | 0 (0-0) | 0.86 (0.3-1.69) | -2.23 (-2.42 to -2.04) |
| Samoa | 1 (0-1) | 2.4 (0.9-4.05) | 1 (0-2) | 1.68 (0.6-3.09) | -0.47 (-0.98 to 0.04) |
| San Marino | 0 (0-0) | 0.46 (0.13-1.03) | 0 (0-0) | 0.26 (0.08-0.59) | -1.94 (-2.03 to -1.84) |
| Sao Tome and Principe | 11 (5-19) | 43.29 (18.36-72.22) | 2 (1-4) | 3.3 (1.28-6.46) | -9.25 (-9.99 to -8.51) |
| Saudi Arabia | 342 (129-607) | 10.48 (3.96-18.62) | 84 (35-151) | 0.83 (0.35-1.49) | -6.92 (-7.32 to -6.51) |
| Senegal | 1475 (728-2141) | 85.74 (42.29-124.42) | 918 (437-1507) | 23.52 (11.21-38.62) | -3.43 (-3.84 to -3.02) |
| Serbia | 33 (11-57) | 1.41 (0.49-2.43) | 7 (2-15) | 0.36 (0.12-0.76) | -4.46 (-4.60 to -4.33) |
| Seychelles | 0 (0-1) | 2.14 (0.8-3.75) | 0 (0-1) | 1.11 (0.4-2.08) | -1.87 (-2.23 to -1.51) |
| Sierra Leone | 659 (286-1096) | 65.62 (28.45-109.2) | 784 (292-1378) | 34.46 (12.83-60.59) | -2.15 (-2.79 to -1.5) |
| Singapore | 4 (2-7) | 0.43 (0.17-0.75) | 1 (0-3) | 0.09 (0.02-0.2) | -5.61 (-6.14 to -5.07) |
| Slovakia | 10 (3-21) | 0.77 (0.23-1.58) | 4 (1-8) | 0.29 (0.08-0.64) | -2.19 (-2.72 to -1.66) |
| Slovenia | 3 (1-6) | 0.55 (0.17-1.16) | 1 (0-3) | 0.3 (0.09-0.7) | -0.82 (-1.23 to -0.42) |
| Solomon Islands | 26 (10-48) | 35.49 (13.19-64.3) | 51 (22-88) | 29.74 (13.02-51.32) | -0.52 (-0.63 to -0.40) |
| Somalia | 5331 (2497-8326) | 314.75 (147.4-491.62) | 8579 (3722-14495) | 177.38 (76.95-299.71) | -1.99 (-2.17 to -1.82) |
| South Africa | 3605 (1453-5970) | 37.34 (15.05-61.84) | 608 (259-961) | 3.92 (1.67-6.21) | -6.31 (-7.70 to -4.89) |
| South Sudan | 1581 (604-2779) | 122.05 (46.64-214.6) | 1611 (649-2952) | 69.4 (27.96-127.15) | -2.13 (-2.27 to -1.99) |
| Spain | 80 (26-162) | 0.83 (0.27-1.68) | 44 (13-93) | 0.44 (0.13-0.94) | -1.17 (-1.47 to -0.87) |
| Sri Lanka | 167 (73-265) | 3.64 (1.59-5.77) | 42 (16-75) | 0.75 (0.29-1.33) | -5.11 (-5.23 to -4.99) |
| Sudan | 7874 (3430-12643) | 168.18 (73.26-270.05) | 2513 (914-4862) | 22.26 (8.09-43.07) | -6.31 (-6.86 to -5.75) |
| Suriname | 7 (3-11) | 6.77 (2.73-11.16) | 5 (2-8) | 3.36 (1.39-5.46) | -2.08 (-2.60 to -1.55) |
| Sweden | 11 (3-25) | 0.56 (0.14-1.23) | 8 (2-19) | 0.39 (0.10-0.86) | -1.11 (-1.40 to -0.82) |
| Switzerland | 16 (6-32) | 0.93 (0.32-1.79) | 12 (4-25) | 0.6 (0.18-1.26) | -1.64 (-1.82 to -1.47) |
| Syrian Arab Republic | 343 (136-538) | 12.45 (4.93-19.52) | 61 (25-112) | 1.58 (0.64-2.87) | -7.04 (-7.36 to -6.73) |
| Taiwan (Province of China) | 34 (9-78) | 0.63 (0.17-1.42) | 12 (3-27) | 0.22 (0.06-0.49) | -3.56 (-3.96 to -3.15) |
| Tajikistan | 110 (46-170) | 9.1 (3.83-14.13) | 88 (37-145) | 3.45 (1.47-5.69) | -3.51 (-3.94 to -3.07) |
| Thailand | 257 (94-452) | 1.61 (0.59-2.83) | 187 (70-321) | 1.15 (0.43-1.97) | 0.05 (-0.73 to 0.84) |
| Timor-Leste | 262 (98-430) | 140.35 (52.59-230.61) | 82 (32-150) | 23.61 (9.18-43.45) | -6.58 (-7.05 to -6.1) |
| Togo | 793 (355-1287) | 92.97 (41.65-150.81) | 479 (193-791) | 22.22 (8.93-36.66) | -4.59 (-5.10 to -4.08) |
| Tokelau | 0 (0-0) | 5.89 (2.27-11.17) | 0 (0-0) | 3.12 (1.24-5.42) | -2.69 (-3.10 to -2.28) |
| Tonga | 1 (0-2) | 4.35 (1.68-7.58) | 0 (0-1) | 1.84 (0.72-3.25) | -2.3 (-2.63 to -1.97) |
| Trinidad and Tobago | 14 (7-21) | 4.65 (2.16-6.86) | 8 (3-13) | 2.44 (1.03-3.86) | -1.36 (-1.80 to -0.91) |
| Tunisia | 143 (56-233) | 7.02 (2.73-11.39) | 31 (11-56) | 1.03 (0.36-1.83) | -5.54 (-5.94 to -5.14) |
| Turkey | 1828 (735-3023) | 12.84 (5.16-21.22) | 251 (99-432) | 1.16 (0.46-1.99) | -7.47 (-8.03 to -6.91) |
| Turkmenistan | 28 (13-46) | 3.16 (1.42-5.23) | 24 (10-41) | 1.93 (0.82-3.25) | -1.61 (-1.91 to -1.32) |
| Tuvalu | 0 (0-1) | 11.96 (4.58-25.89) | 0 (0-0) | 4.52 (1.81-8.54) | -2.78 (-3.30 to -2.26) |
| Uganda | 3603 (1595-5829) | 93.63 (41.45-151.47) | 2732 (1087-4809) | 26.24 (10.44-46.18) | -4.41 (-4.94 to -3.89) |
| Ukraine | 203 (72-356) | 1.6 (0.57-2.81) | 62 (20-124) | 0.62 (0.19-1.23) | -2.20 (-2.62 to -1.78) |
| United Arab Emirates | 9 (3-16) | 2.53 (1-4.69) | 9 (4-16) | 0.51 (0.21-0.95) | -4.64 (-4.87 to -4.41) |
| United Kingdom | 96 (29-199) | 0.68 (0.21-1.4) | 73 (22-149) | 0.47 (0.14-0.97) | -0.50 (-0.79 to -0.21) |
| United Republic of Tanzania | 4766 (2622-6473) | 79.43 (43.69-107.87) | 3953 (1685-6249) | 26.61 (11.34-42.06) | -3.3 (-3.88 to -2.72) |
| United States of America | 734 (253-1428) | 1.1 (0.38-2.13) | 457 (146-876) | 0.6 (0.19-1.16) | -2.26 (-2.38 to -2.13) |
| United States Virgin Islands | 1 (0-1) | 2.21 (0.83-4.07) | 0 (0-0) | 0.83 (0.31-1.51) | -2.82 (-2.93 to -2.71) |
| Uruguay | 59 (26-90) | 7.83 (3.45-11.99) | 21 (8-34) | 2.49 (1-4.13) | -3.36 (-3.67 to -3.05) |
| Uzbekistan | 245 (117-358) | 4.99 (2.4-7.31) | 124 (59-207) | 1.4 (0.66-2.32) | -3.23 (-3.75 to -2.71) |
| Vanuatu | 4 (2-7) | 11.32 (4.38-20.69) | 6 (2-10) | 7.39 (3.16-12.57) | -1.53 (-1.69 to -1.36) |
| Venezuela (Bolivarian Republic of) | 739 (329-1110) | 15.26 (6.79-22.94) | 400 (160-679) | 5.83 (2.32-9.9) | -2.80 (-3.40 to -2.20) |
| Viet Nam | 407 (144-710) | 2.39 (0.84-4.16) | 139 (46-283) | 0.54 (0.18-1.11) | -4.30 (-4.68 to -3.92) |
| Yemen | 3451 (1693-5512) | 126 (61.8-201.22) | 2527 (1128-4360) | 30.24 (13.5-52.18) | -5.22 (-5.51 to -4.93) |
| Zambia | 1197 (523-1920) | 65.09 (28.45-104.38) | 971 (386-1733) | 19.73 (7.84-35.23) | -4.20 (-4.65 to -3.74) |
| Zimbabwe | 1617 (718-2556) | 67.24 (29.87-106.31) | 2063 (832-3527) | 50.81 (20.48-86.87) | 1.28 (0.06 to 2.52) |

MSMIs, maternal sepsis and other maternal infections; ASR, age-standardized rate; EAPC, estimated annual percentage change; CI, confidence interval.
